# Validation of a brief image elicitation task as an indicator of subjective wellbeing in the general population

**DOI:** 10.3389/fpubh.2024.1435144

**Published:** 2024-10-22

**Authors:** J. David Pincus

**Affiliations:** Research and Development Department, Leading Indicator Systems, Boston, MA, United States

**Keywords:** wellbeing, emotional wellbeing, emotional needs, image-elicitation, short form, thriving, happiness, wellness

## Abstract

**Background:**

A novel image-based method (AgileBrain) demonstrates construct validity as a measure of wellbeing in the general working adult population.

**Method:**

Analysis of data from four large nationally representative samples of American full-time workers employed by mid-to-large size companies conducted in November 2021 (*n* = 812), May 2022 (*n* = 810), June 2023 (*n* = 986), and January 2024 (*n* = 1,179).

**Results:**

Across all four studies, AgileBrain demonstrates convergent validity across multiple established indicators of subjective wellbeing including the Patient Health Questionnaire (PHQ-9), Center for Epidemiological Studies Depression Scale (CESD-10), Generalized Anxiety Disorder Scale (GAD-7), Neuroticism (BFI-S), UCLA Loneliness Scale, Perceived Stress Scale (PSS-10), Coping Styles (Brief COPE-28), self-reported diagnosed neurodiversity conditions and symptoms, and trauma history.

**Conclusion:**

Results across these studies suggest that AgileBrain is useful as a screening tool for detecting compromised wellbeing in terms of construct validity. Given strong preferences for brief, gamified assessments, the validity advantages stemming from less consciously controllable responses, and the statistical advantages of measures associated with high response rates and normal distributions, AgileBrain emerges as strong tool for assessing subjective wellbeing at the population level and offers a promising approach to monitoring treatment effectiveness.

## 1 Background

A novel method for measuring the presence and intensity of emotional needs has recently been proposed (1). The method, entitled *AgileBrain*, utilizes rapid-exposure image selection to capture affective responses by bypassing cognitive barriers. This methodological innovation is designed as a gamified, engaging assessment that requires only 3 min of user’s attention, resulting in enhanced completion rates and willingness to engage in repeated assessments over time, which are both critical for monitoring population wellbeing. The use of images rather than text also enhances the generalizability of the model across different settings and cultures by reducing language-based biases. Image presentation and response window latencies adhere to the neurological time course described by Damasio and colleagues for capturing emotional responses before cognition can bias or filter response (2, 3).

The context within which this new method is being introduced is a broad and deep mental health crisis in the United States, and globally, which has prompted a concerted response from governmental and non-governmental organizations to address this challenge. According to the Centers for Disease Control, between August 2020 and February 2021, the proportion of adults experiencing recent symptoms of common mental health disorders (CMDs) such as anxiety or depressive disorders rose from 36.4 to 41.5%. Concurrently, the percentage of adults who reported unmet needs for mental health care also increased, from 9.2 to 11.7%. Notably, these increases were most pronounced among adults aged 18–29 years (4).

Complicating matters for public health officials, there is growing concern about declining survey response rates for monitoring population wellbeing. The Association of American Medical Colleges, for example, reports a consistent annual decline in response rates to their health care surveys. An analysis of national data also shows a decrease in the overall patient response rate, dropping from 33% in 2008 to 26% in 2017 (18). A particularly important consequence of decreasing response rate is increasing non-response bias, the systematic tendency of certain types of individuals or subgroups to respond at lower rates than others, causing the results to be non-representative, thereby compromising the reliability and validity of wellbeing survey results. Investigators have called for new approaches to monitoring population wellbeing that would be (1) shorter to complete, (2) easier to complete by respondents with lower levels of health literacy, and (3) take the form of scalable, self-directed digital experiences (18). By utilizing a gamified rapid image exposure protocol, AgileBrain accomplishes all three of these goals. In addition to these benefits, AgileBrain avoids common cognitive biases known to plague traditional surveys such as response acquiescence, social desirability bias, and response style. Further, AgileBrain is unique in its ability to indirectly assess latent emotional states, which are notoriously difficult to measure because they largely operate outside conscious awareness.

Qualitative studies have indicated that mental health service users and their clinicians prefer AgileBrain over other commonly used wellbeing surveys (5). Accordingly, AgileBrain is now being used in clinical settings at universities and within the U.S. military on a pilot basis, often in conjunction with traditional measures such as the PHQ-9 and GAD-7. Investigating the effectiveness of AgileBrain as a monitoring and outcome measure in individuals with common mental disorders (CMD) has become crucial. This approach aims to facilitate comparisons of methodologies in large scale general populations. Research conducted in diverse environments indicates that these tools are valid within clinical and non-clinical coaching settings. However, their comparative effectiveness alongside established clinical assessments and other potential indicators of overall wellbeing remains unexplored.

### 1.1 Defining emotional wellbeing

A recent literature review (6) has demonstrated that subjective wellbeing as a construct can be thought of as a summary state of human need fulfillment. To the extent that needs are met, we feel well; to the extent that our needs are unmet, we feel unwell. Through a comprehensive mapping of SWB concepts and assessment items, this concept can be decomposed into degrees of fulfillment of 12 clearly defined emotional needs, each having extensive literatures of their own (7). The U.S. Surgeon General’s recently proposed atheoretical model of SWB has been shown to closely align with this theory-based multidimensional model of SWB (6).

SWB is inherently complex, representing an ever-changing summary internal milieu representing 12 categories of need fulfillment, each having promotion and prevention needs. Accordingly, attempts to reduce SWB to simple measures of happiness, thriving, contentment, etc. provide only limited, superficial readings that ignore the delicate interplay of needs and resources below the surface. We argue that SWB is a sufficiently broad concept that it should reflect associations with a wide range of facets of wellbeing, with significant but modest correlations with a host of indicators, but not should overlap substantially with any one of these indicators.

Accordingly, we evaluated a wide variety of SWB indicators alongside AgileBrain to test this hypothesis. These included clinical assessments of depression and anxiety, loneliness, stress levels, coping styles, the personality trait of neuroticism, neurodiversity diagnoses and symptoms, and self-reported trauma. By intentionally casting a wide net, we sought to demonstrate patterns of SWB differences that cut across multiple conceptual systems of psychology, psychiatry, and public health. This study focuses on evaluating the construct validity of AgileBrain as an SWB indicator in general populations in comparison multiple indicators that theoretically ought to be associated with different levels of SWB. Comparisons are made between the AgileBrain profiles for the general population and those reporting different levels of each of the above conditions.

## 2 Materials and methods

### 2.1 Participants

Surveys were administered to four^1^ population-representative samples of US-based employees working full time who were recruited by professional research panel companies, to participate in an 18-min anonymized survey, for which they were compensated using the panels’ compensation systems. Ethics approval and consent to participate: All participants are members of commercial survey panels, InnovateMR, LLC and Prodege, LLC, which are governed by their own ethical review processes and guidelines. Accordingly, there was no need for separate ethics approval. Waves were collected in November 2021, May 2022, June 2023, and January 2024. Participants were drawn from population-representative samples of American employees who are currently working full time for companies with at least 20 employees, in proportion to the Bureau of Labor Statistics’ distributions of employment by employer size. This step ensures that the samples are representative of the US population of similarly situated workers. Each wave of this national survey has a statistical confidence level of 95 percent with margins of error ranging from +/− 2.85 to 3.44 percent. The resulting samples are generally representative of US full-time workers as estimated by the U.S. Bureau of Labor Statistics Current Population Survey, corresponding to BLS distributions for sex, age, and race (8).

### 2.2 Measures

We employed three standard clinical and public health outcome measures:

- Patient Health Questionnaire (PHQ-9). The PHQ-9 is a nine-item self-report tool used to screen for the presence and severity of depression. It assesses the frequency of depressive symptoms over the past 2 weeks, providing a score that helps guide diagnosis and treatment decisions (9).
- Center for Epidemiological Studies Depression Scale (CESD-10). The CESD-10 is a 10-item questionnaire designed to measure depressive symptoms in the general population. It is used for screening and research purposes to identify individuals at risk for depression (10, 11).
- Generalized Anxiety Disorder Scale (GAD-7). The GAD-7 is a seven-item scale used to identify and measure the severity of generalized anxiety disorder. It evaluates how often patients experience anxiety-related symptoms in the past 2 weeks, aiding in clinical assessment and management (12).

Comparisons are also made with commonly used measures of psychological distress:

- UCLA Loneliness Scale (UCLA-3). The UCLA Loneliness Scale (3-item) is a short-form version of the original UCLA Loneliness Scale, used to assess subjective feelings of loneliness and social isolation. It consists of three questions that gauge how often individuals feel disconnected from others (13).
- Perceived Stress Scale (PSS-10). The PSS-10 is a 10-item self-report instrument that measures the degree to which situations in one’s life are perceived as stressful. It evaluates the extent of stress experienced over the past month, providing insights into an individual’s stress levels and coping abilities (14).
- Adaptive and Maladaptive Coping Styles (Brief COPE-28). The Brief COPE-28 is a 28-item questionnaire designed to assess a wide range of coping strategies that individuals use in response to stress. It covers various coping mechanisms, including problem-focused, emotion-focused, and dysfunctional coping styles (15, 21).
- Neuroticism dimension of the Big Five Personality Inventory (BFI-15). The Neuroticism scale of the BFI-15 measures an individual’s tendency to experience negative emotions such as anxiety, anger, and depression. It assesses the stability of emotions and the extent to which individuals perceive the world as threatening, with higher scores indicating greater emotional instability and sensitivity to stress (16).

Comparisons are also made with self-reported psychological conditions that ought to correlate with SWB:

- Formally diagnosed neurodiversity: We measured six types of diagnosed neurodiversity: Autism spectrum (ASD); Attention deficit disorder (ADD or ADHD); Developmental Coordination Disorder (Dyspraxia); Obsessive Compulsive Disorder (OCD); other provided by participants (primarily bipolar disorder and post-traumatic stress disorder); or none of these (20).
- Experienced ADD/ADHD symptoms: We measured eight types of symptoms: Impulsiveness; disorganization and problems prioritizing; poor time management; problems focusing on, or completing, tasks; trouble multitasking; excessive activity or restlessness; difficulty coping with stress; or none of these (APA, 2022).
- Traumatic experiences: We measured six types of trauma: current trauma, which involves ongoing distressing events; acute trauma, resulting from a single, isolated event; repetitive trauma from repeated exposure to similar traumatic events; complex trauma from multiple, varied, and prolonged traumatic events; and developmental trauma, which occurs during critical periods of childhood development due to chronic exposure to trauma; and no trauma, indicating the absence of significant traumatic experiences (APA, 2022).

### 2.3 Procedure

Four large-scale surveys included the image protocol to assess employee emotional needs. The psychometric validity and reliability of AgileBrain, along with descriptions of its protocols, have been previously documented (1). These questionnaires also included the wellbeing indicators listed above for establishing construct validity, grouped below by the wave of research:

- November 2021: PSS-10, Brief COPE-28
- May 2022: UCLA-3
- June 2023: CESD-10, Trauma, BFI-15, Neurodiversity
- January 2024: PHQ-9, GAD-7

### 2.4 Study design and data analyses

This is a descriptive, correlational study with a cross-sectional design. Analyses were conducted using SPSS 24.0.

## 3 Results

### 3.1 Descriptives

The mean ages, genders, and races are consistent with each other and with the Bureau of Labor Statistics (Table 1).

**Table 1:** Sample characteristics.

	Bureau of labor statistics^1^	November 2021	May 2022	June 2023	January 2024
Sample size	60,000	812	810	986	1,177
Margin of error at 95% CI	+/− < 1%	+/−3.44%	+/−3.44%	+/−3.12%	+/−2.85%
Response rate	71%	28%	25%	26%	23%
Sex					
Male	56.3%	57.1%	55.4%	56.6%	57.1%
Female	43.7%	42.9%	44.6%	43.4%	42.9%
Age					
18–19	1.0%	*	1.0%	0.8%	7.8%
20–24	7.2%	*	7.0%	7.4%	
25–34	25.1%	30.6%	25.6%	23.9%	26.3%
35–44	24.4%	30.6%	24.9%	24.4%	26.6%
45–54	23.4%	38.0%	23.4%	23.8%	26.5%
55+	19.0%	*	18.1%	19.6%	(55–64) 12.8%
Race					
Asian (non-Hispanic)	6.6%	7.7%	6.9%	6.9%	8.6%
Black (non-Hispanic)	11.8%	11.6%	12.9%	12.9%	12.9%
Hispanic/Latino	18.5%	21.1%	17.8%	19.5%	21.1%
White (non-Hispanic)	63.0%	67.9%	70.2%	68.5%	71.7%

^1^U. S. Bureau of Labor Statistics (2023). Labor Force Statistics from the Current Population Survey. Demographic characteristics of U.S. workers, employed, usually work full-time, by age, sex, and race. *The sample collected in November 2021 was limited to ages 25–54.

**Figure 1:**
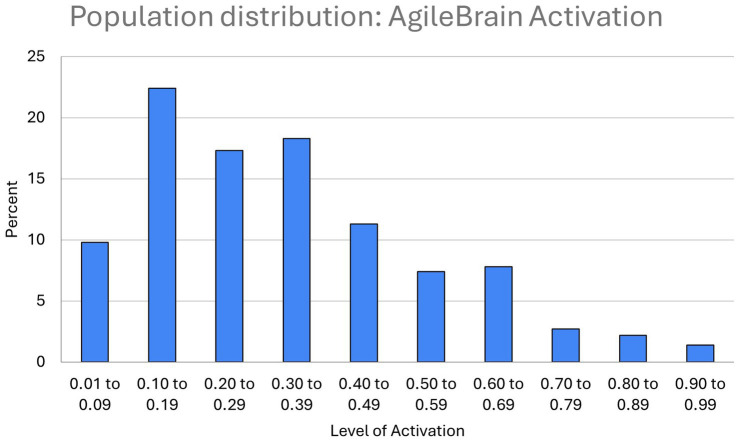
Population distribution of AgileBrain activation scores.

**Figure 2:**
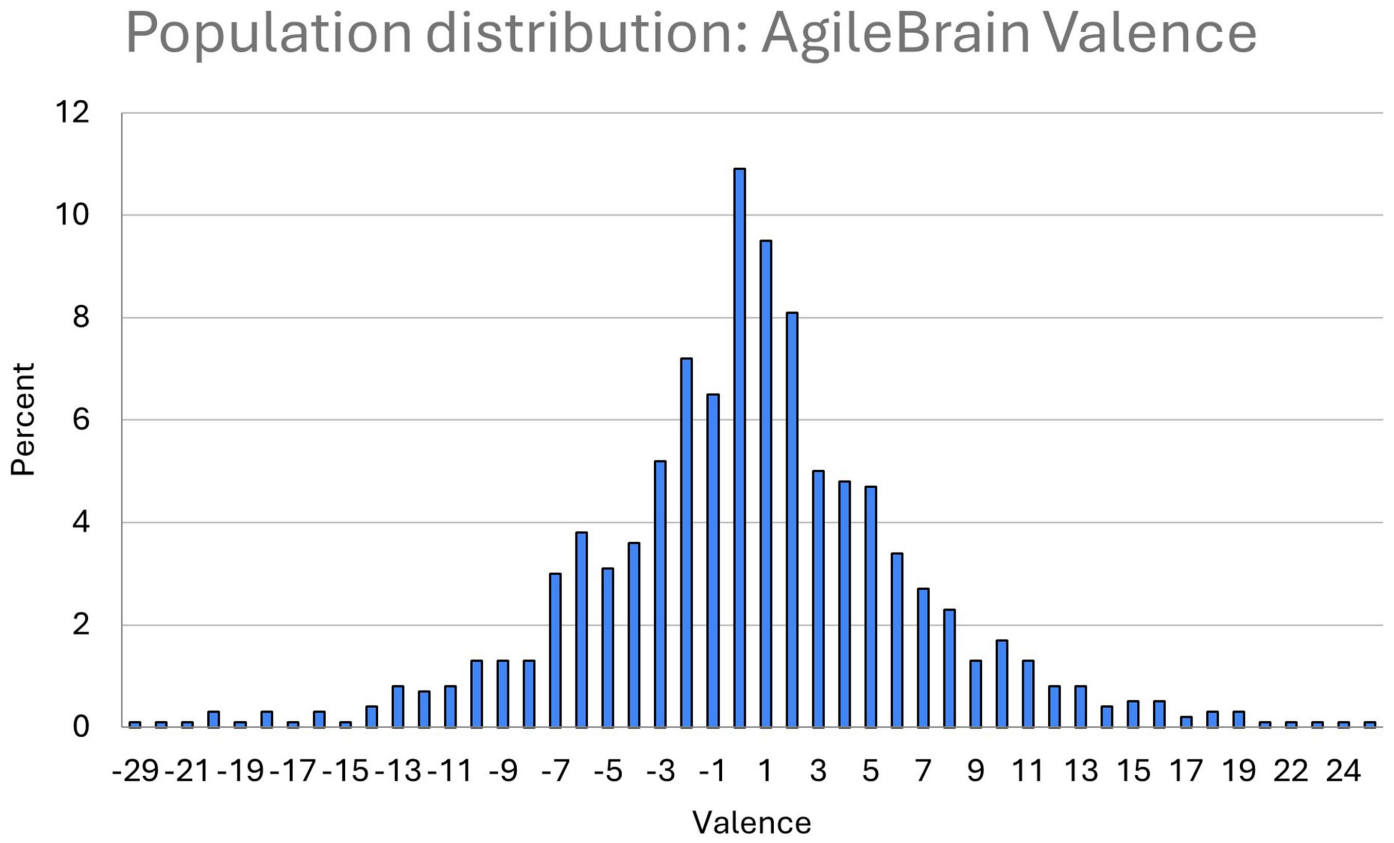
Population distribution of AgileBrain valence scores.

**Figure 3:**
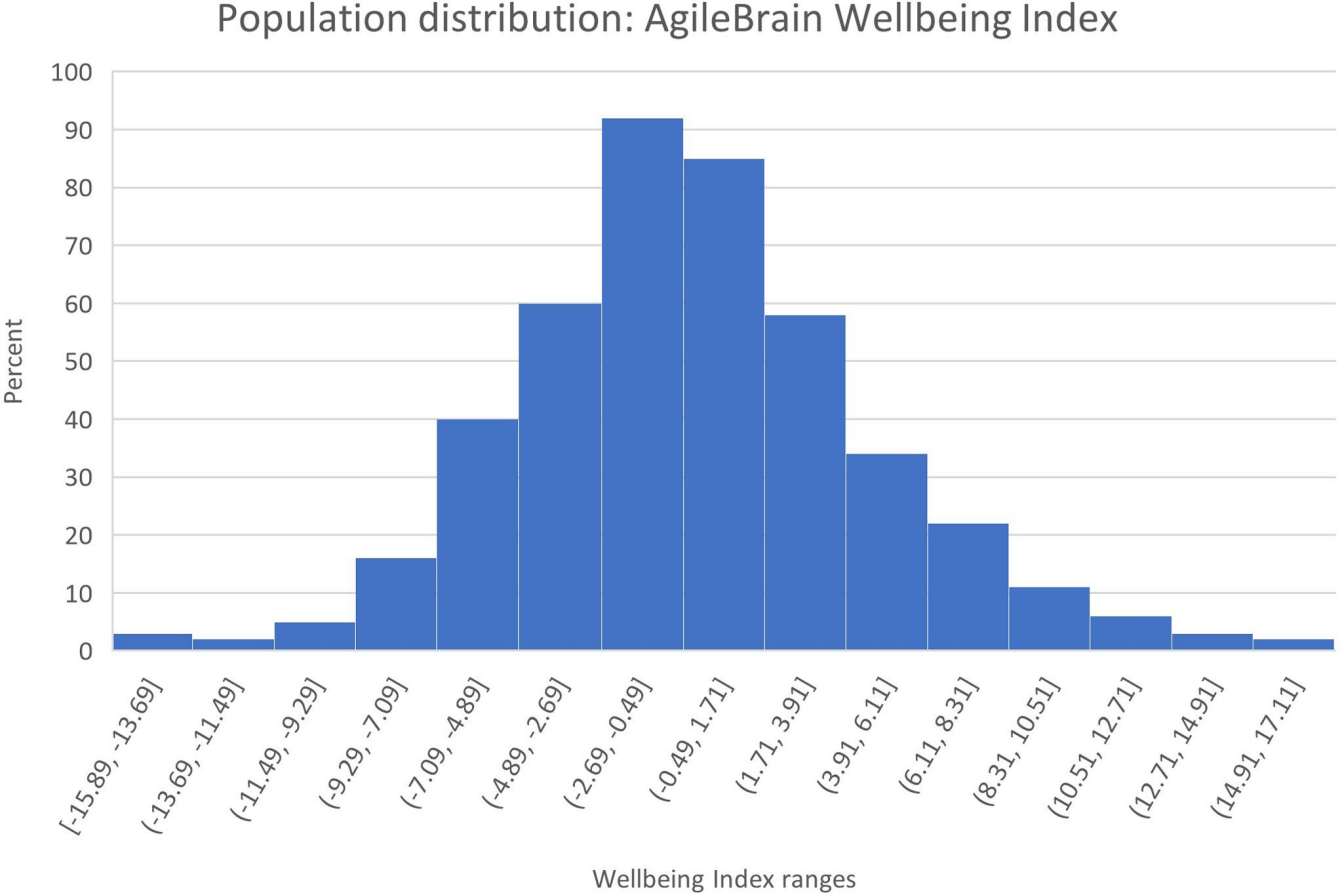
Population distribution of AgileBrain wellbeing index scores.

Population distributions for the three summary-level AgileBrain measures are presented in Figures 1–3. AgileBrain produces three overall scores as well as component scores. The three overall scores are (1) emotional activation or intensity of unmet needs (mean = 0.334, SD = 0.161; Figure 1), (2) emotional valence (mean = 0.436, SD = 6.21; Figure 2), and (3) the product of (1 - activation decimal) and valence into a summary wellbeing index (mean = −0.191, SD = 3.835; Figure 3). For 6.3% (*n* = 74) activation scores were at or above the theoretical 1 SD cut point for compromised mental wellbeing of 0.64. For 14.9% (*n* = 173) valence scores were at or below the theoretical 1 SD cut point for compromised mental wellbeing of-6. For 7.4% (*n* = 87) valence scores were at or below the theoretical 1 SD cut point for compromised mental wellbeing of −4.6. AgileBrain scores for (1) activation are similarly positively skewed as more people in the general population tend to be emotionally settled at a given point time than unsettled or activated. AgileBrain scores are normally distributed in the general population for (2) valence and (2) wellbeing index.

PHQ-9, CESD-10, GAD-7, UCLA-3, coping styles of avoidance or self-blame, reporting a neurodiversity diagnosis, and history of trauma are not normally distributed; all are positively skewed distributions, as expected, as most people in the general population do not suffer from these conditions or tendencies and, accordingly, produce lower scores (Supplementary Figures S1, S3, S7, S9, S20, S24, S26).

For 26.0% (*n* = 306), PHQ-9 scores met or exceeded the clinical threshold for depression of 10 (mean = 6.39, SD = 6.10; Supplementary Figure S2). For 29.0% (*n* = 299), CESD-10 scores met or exceeded the threshold for moderate or severe depression (mean = 11.37, SD = 6.51; Supplementary Figure S3). For 18.8% (*n* = 222) of participants, GAD-7 scores exceeded the clinical threshold for anxiety of 10 (mean = 5.33, SD = 5.60; Supplementary Figure S5). PHQ-9 scores and GAD-7, which were both included in the January 2024 research wave, their correlation could be measured; PHQ-9 and GAD-7 scores overlap substantially with a Pearson correlation of 0.848 (*p* < 0.0001).

For 36.5% (*n* = 360) of participants, UCLA-3 scores exceed the clinical threshold for loneliness (mean = 4.92, SD = 1.85; Supplementary Figure S7). For 25.9% (*n* = 177), PSS-10 scores exceed the clinical threshold of 27 for high levels of stress (mean = 22.88, SD = 6.69; Supplementary Figure S8).

There are no established clinical thresholds for the coping styles measures; the percentage exceeding 1 SD is reported instead. For Avoidant coping, 19.5% (*n* = 133) exceeded 1 SD (mean = 12.08, SD = 5.23; Supplementary Figure S9). For Problem-Focused coping, 12.5% (*n* = 85) exceeded 1 SD (mean = 11.06, SD = 2.92; Supplementary Figure S10). For Social Support-based coping, 16.0% (*n* = 108) exceeded 1 SD (mean = 9.84, SD = 3.36; Supplementary Figure S12). For Spirituality-based coping, 19.0% (*n* = 130) exceeded 1 SD (mean = 10.25, SD = 3.21; Supplementary Figure S14). For Distraction-based coping, 10.8% (*n* = 74) exceeded 1 SD (mean = 5.22, SD = 1.59; Supplementary Figure S16). For Externalization-based coping, 13.0% (*n* = 90) exceeded 1 SD (mean = 9.54, SD = 3.20; Supplementary Figure S18). For Self-Blame-based coping, 10.0% (*n* = 68) exceeded 1 SD (mean = 4.53, SD = 1.94; Supplementary Figure S20). For Acceptance-based coping, 13.0% (*n* = 90) exceeded 1 SD (mean = 5.42, SD = 1.67; Supplementary Figure S21).

Similarly, there is no established clinical threshold for the personality trait of neuroticism. 15.3% (*n* = 158) exceeded 1 SD (mean = 2.90, SD = 0.94; Supplementary Figure S23).

With respect to diagnosed neurodiversity, 34.0% report receiving at least one such diagnosis. In order of prevalence, we estimate the following prevalence: ADD/ADHD (19.50%, *n* = 201), Obsessive Compulsive Disorder (9.60%, *n* = 99), Developmental Coordination Disorder (2.62%, *n* = 27), Autism Spectrum Disorder (2.62%, *n* = 27), Bipolar Disorder (0.97%, *n* = 10), and PTSD (0.87%, *n* = 9; Supplementary Figure S24).

Regarding symptom prevalence, 78.0% report at least one ADD/ADHD symptom. In order of prevalence, we estimate the following levels: difficulty coping with stress (34.2%, *n* = 678), transitioning between tasks (32.2%, *n* = 332), restlessness (31.4%, *n* = 324), disorganization (26.4%, *n* = 272), impulsivity (26.0%, *n* = 268), poor time management (25.8%, *n* = 266), and difficulty multi-tasking (19.7%, *n* = 203; Supplementary Figure S25).

The majority, 57.4% of participants (*n* = 592), reported having experienced at least one form of trauma. Acute trauma (32.4%, *n* = 334), complex trauma (24.2%, *N* = 250), repetitive trauma (20.1%, *n* = 207), developmental trauma (16.7%, *n* = 172), current trauma (4.40%, *n* = 45; Supplementary Figure S26).

### 3.2 Correlation between measures

**Figure 4:**
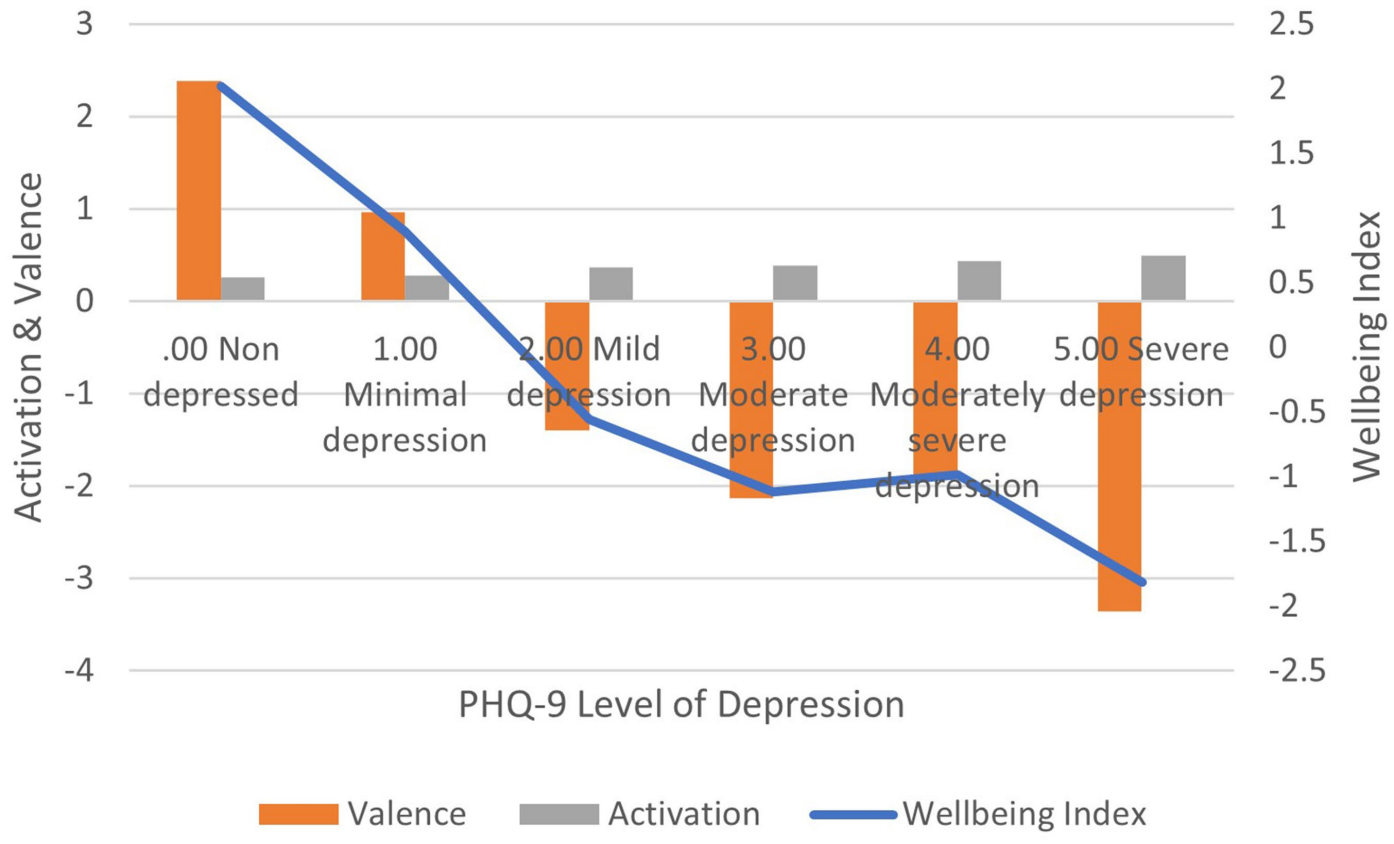
AgileBrain valence, activation, and wellbeing index scores by PHQ-9 levels of depression.

Correlation between PHQ-9 scores and: (1) activation is positive and significant (*r* = 0.301, *p* < 0.01); (2) valence is negative and significant (*r* = −0.291, *p* < 0.01); (3) wellbeing index is negative and significant (*r* = −0.210, *p* < 0.01) (Figure 4). In terms of effect size for the wellbeing index, Cohen’s *d* = 2.93, an extremely large effect.

**Figure 5:**
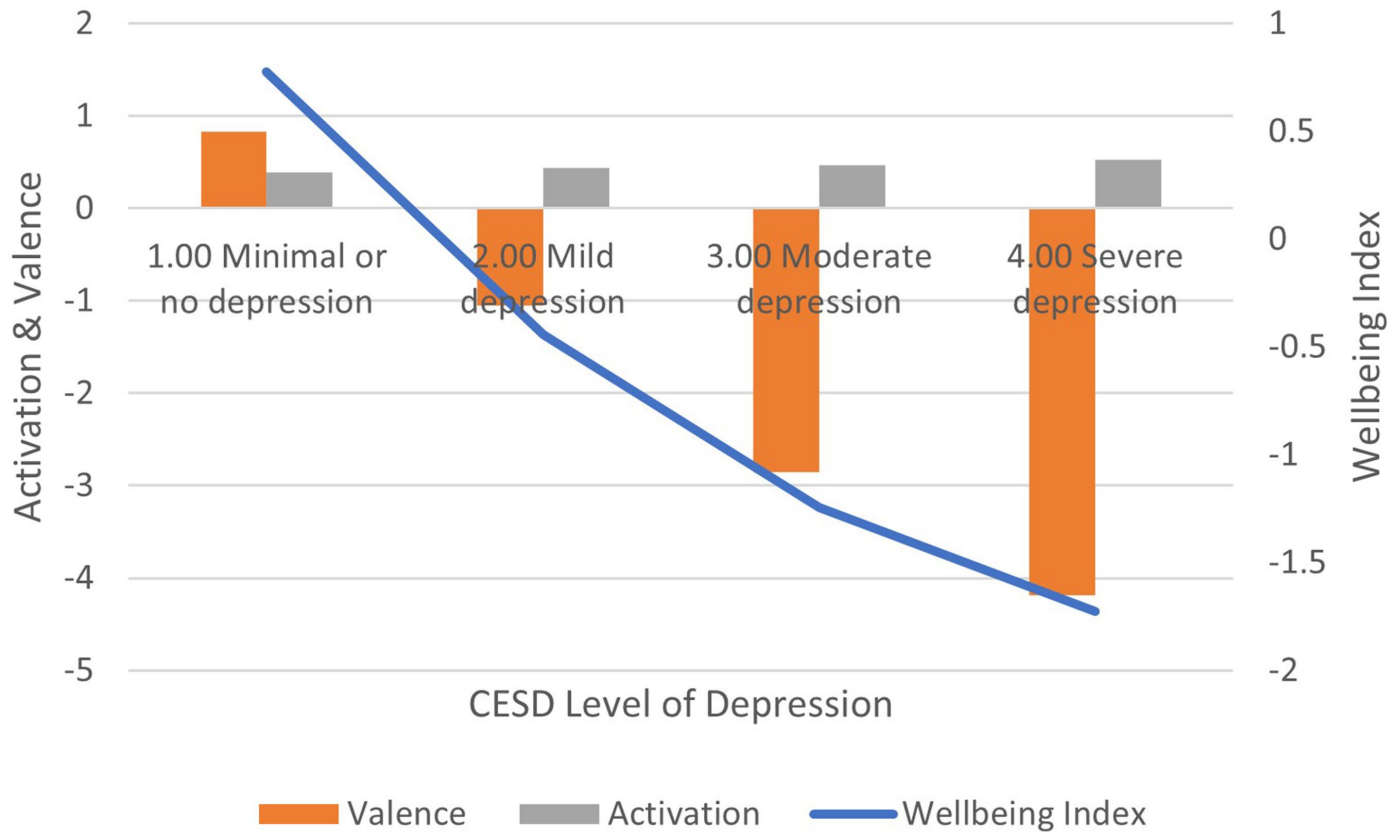
AgileBrain valence, activation, and wellbeing index scores by CESD-10 levels of depression.

Correlation between CESD-10 scores and: (1) activation is positive and significant (*r* = 0.206, *p* < 0.01); (2) valence is negative and significant (*r* = −0.272, *p* < 0.01); (3) wellbeing index is negative and significant (*r* = −0.250, *p* < 0.01) (Figure 5). In terms of effect size for the wellbeing index, Cohen’s *d* = 2.63, an extremely large effect.

**Figure 6:**
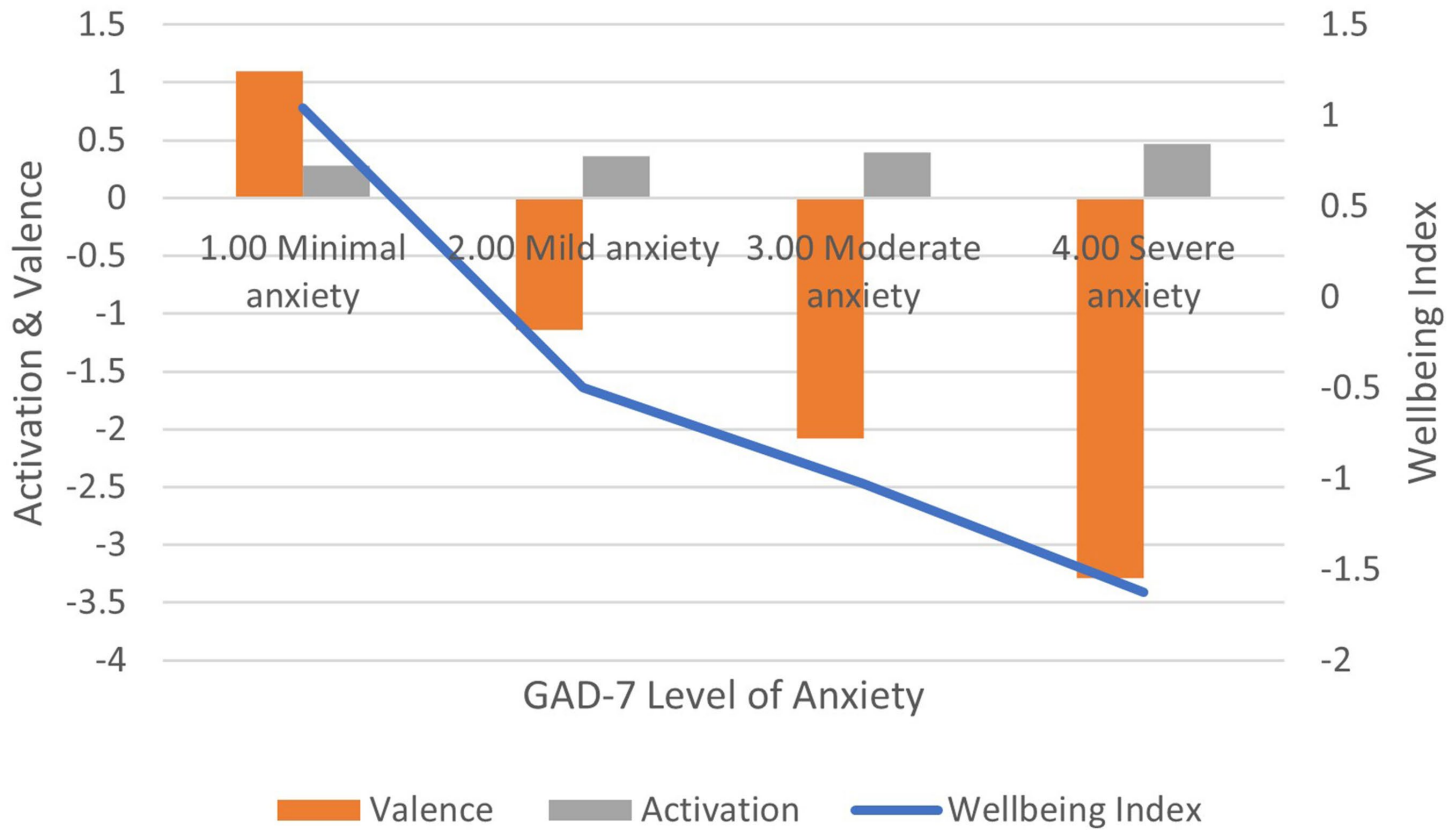
AgileBrain valence, activation, and wellbeing index scores by GAD-7 levels of anxiety.

Correlation between GAD-7 scores and: (1) activation is positive and significant (*r* = 0.308, *p* < 0.01); (2) valence is negative and significant (*r* = −0.309, *p* < 0.01); (3) wellbeing index is negative and significant (*r* = −0.242, *p* < 0.01) (Figure 6). In terms of effect size for the wellbeing index, Cohen’s *d* = 2.70, an extremely large effect.

**Figure 7:**
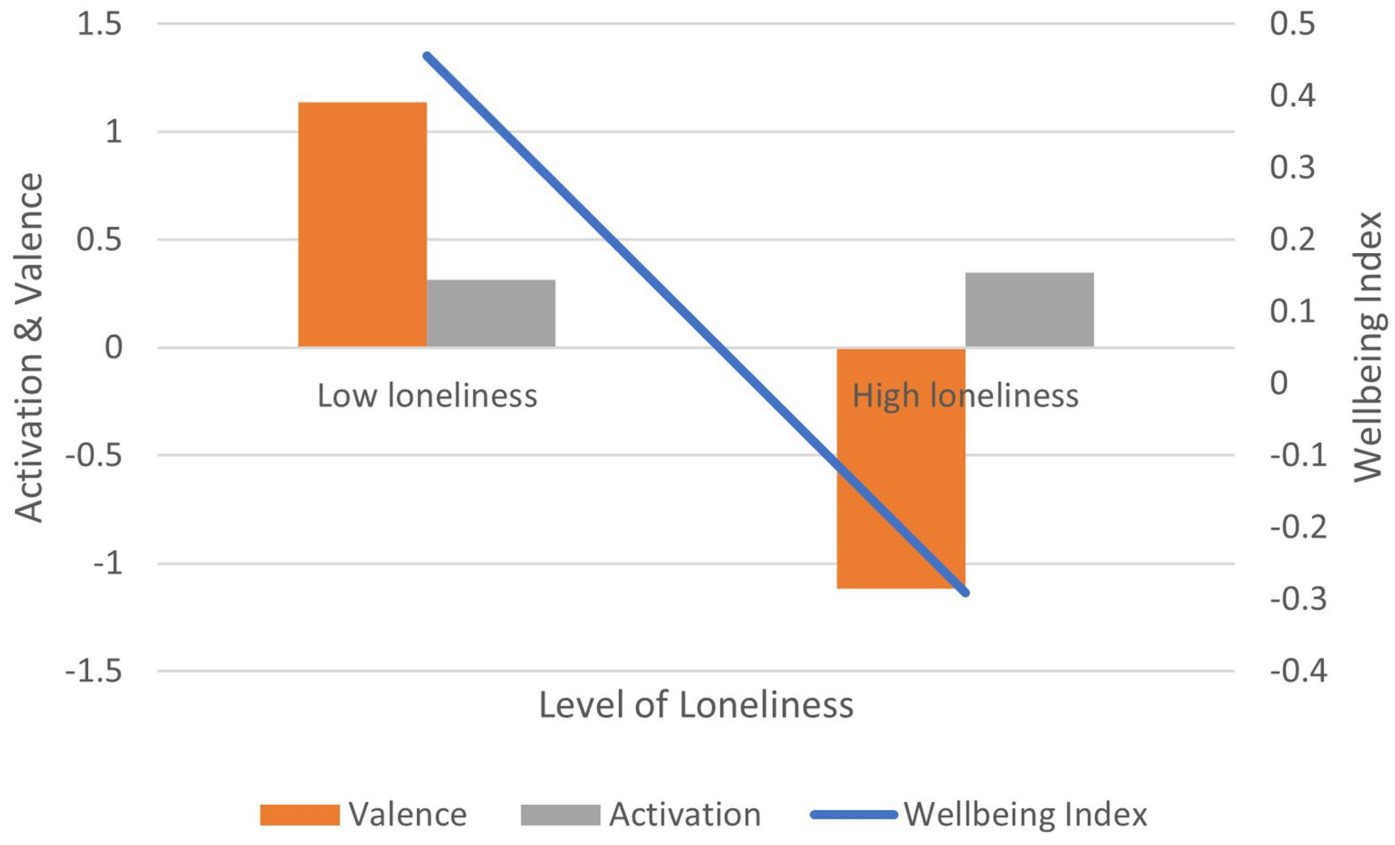
AgileBrain valence, activation, and wellbeing index scores by UCLA loneliness categories.

Correlation between UCLA-3 loneliness scores and: (1) activation is positive and significant (*r* = 0.136, *p* < 0.01); (2) valence is negative and significant (*r* = −0.153, *p* < 0.01); (3) wellbeing index is negative and significant (*r* = −0.164, *p* < 0.01) (Figure 7). In terms of effect size for the wellbeing index, Cohen’s *d* = 2.02, an extremely large effect.

**Figure 8:**
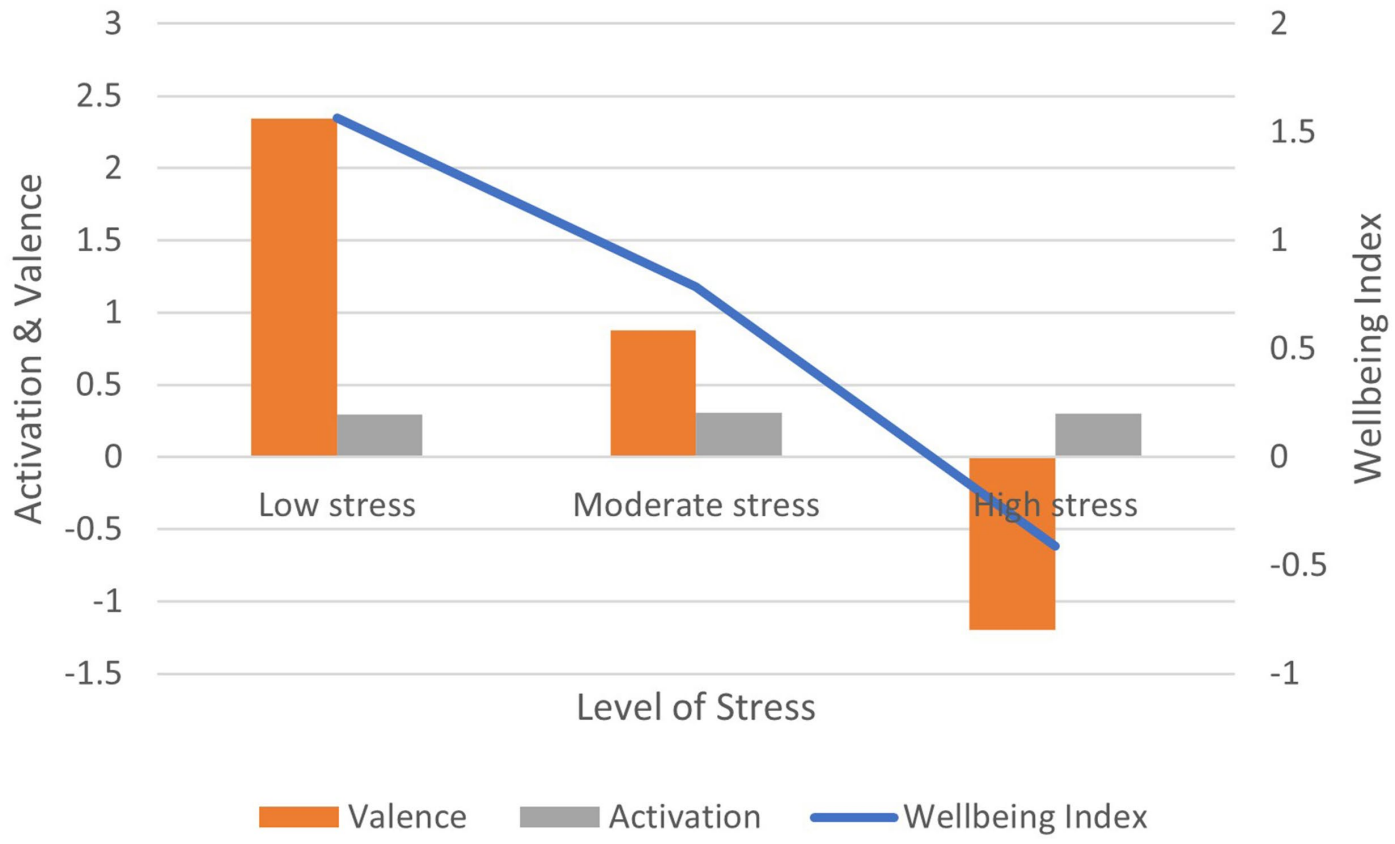
AgileBrain valence, activation, and wellbeing index scores by PSS-10 stress levels.

Correlation between the PSS-10 stress scores and: (1) activation is non-significant (*r* = 0.021, ns); (2) valence is negative and significant (*r* = −0.148, *p* < 0.01); (3) wellbeing index is negative and significant (*r* = −0.138, *p* < 0.01) (Figure 8). In terms of effect size for the wellbeing index, Cohen’s *d* = 2.44, an extremely large effect.

**Figure 9:**
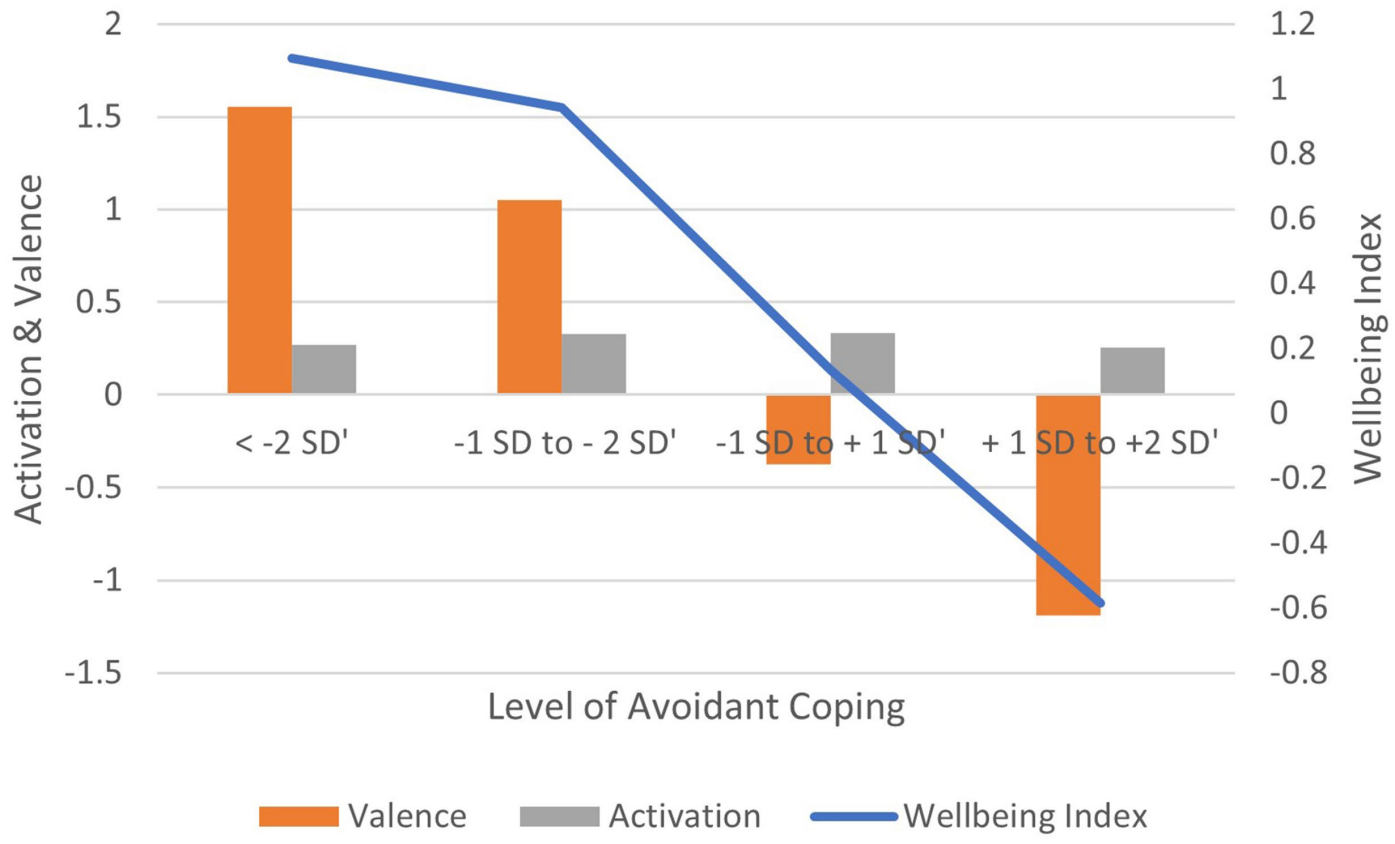
AgileBrain valence, activation, and wellbeing index scores by levels of avoidant coping.

Correlation between Avoidant coping scores and: (1) activation is non-significant (*r* = −0.041, ns); (2) valence is negative and significant (*r* = −0.163, *p* < 0.01); (3) wellbeing index is negative and significant (*r* = −0.140, *p* < 0.01) (Figure 9). In terms of effect size for the wellbeing index, Cohen’s d = 2.80, an extremely large effect.

Correlation between Problem-focused coping scores and: (1) activation is non-significant (*r* = 0.042, ns); (2) valence is negative and significant (*r* = −0.087, *p* < 0.05); (3) wellbeing index is negative and significant (*r* = −0.077, *p* < 0.05) (Supplementary Figure S11). In terms of effect size for the wellbeing index, Cohen’s *d* = 1.91, an extremely large effect.

Correlation between Social Support-based coping scores and: (1) activation is non-significant (*r* = −0.073, ns); (2) valence is non-significant (*r* = −0.052, ns); (3) wellbeing index is non-significant (*r* = −0.035, ns) (Supplementary Figure S13). In terms of effect size for the wellbeing index, Cohen’s *d* = 1.43, an extremely large effect.

Correlation between Spiritual coping scores and: (1) activation is non-significant (*r* = −0.009, ns); (2) valence is non-significant (*r* = 0.016, ns); (3) wellbeing index is non-significant (*r* = −0.001, ns) (Supplementary Figure S15). In terms of effect size for the wellbeing index, Cohen’s *d* = − 1.82, an extremely large effect. Note that Spiritual coping is the only instance in the present study where more engagement in the practice is associated with improved wellbeing (hence, the negative effect size).

Correlation between Distraction-based coping scores and: (1) activation is non-significant (*r* = 0.074, ns); (2) valence is negative and significant (*r* = −0.122, *p* < 0.01); (3) wellbeing index is negative and significant (*r* = −0.114, *p* < 0.01) (Supplementary Figure S17). In terms of effect size for the wellbeing index, Cohen’s *d* = 1.52, an extremely large effect.

Correlation between Externalized coping scores and: (1) activation is non-significant (*r* = −0.002, ns); (2) valence is negative and significant (*r* = −0.158, *p* < 0.01); (3) wellbeing index is negative and significant (*r* = −0.147, *p* < 0.01) (Supplementary Figure S19). In terms of effect size for the wellbeing index, Cohen’s *d* = 2.35, an extremely large effect.

**Figure 10:**
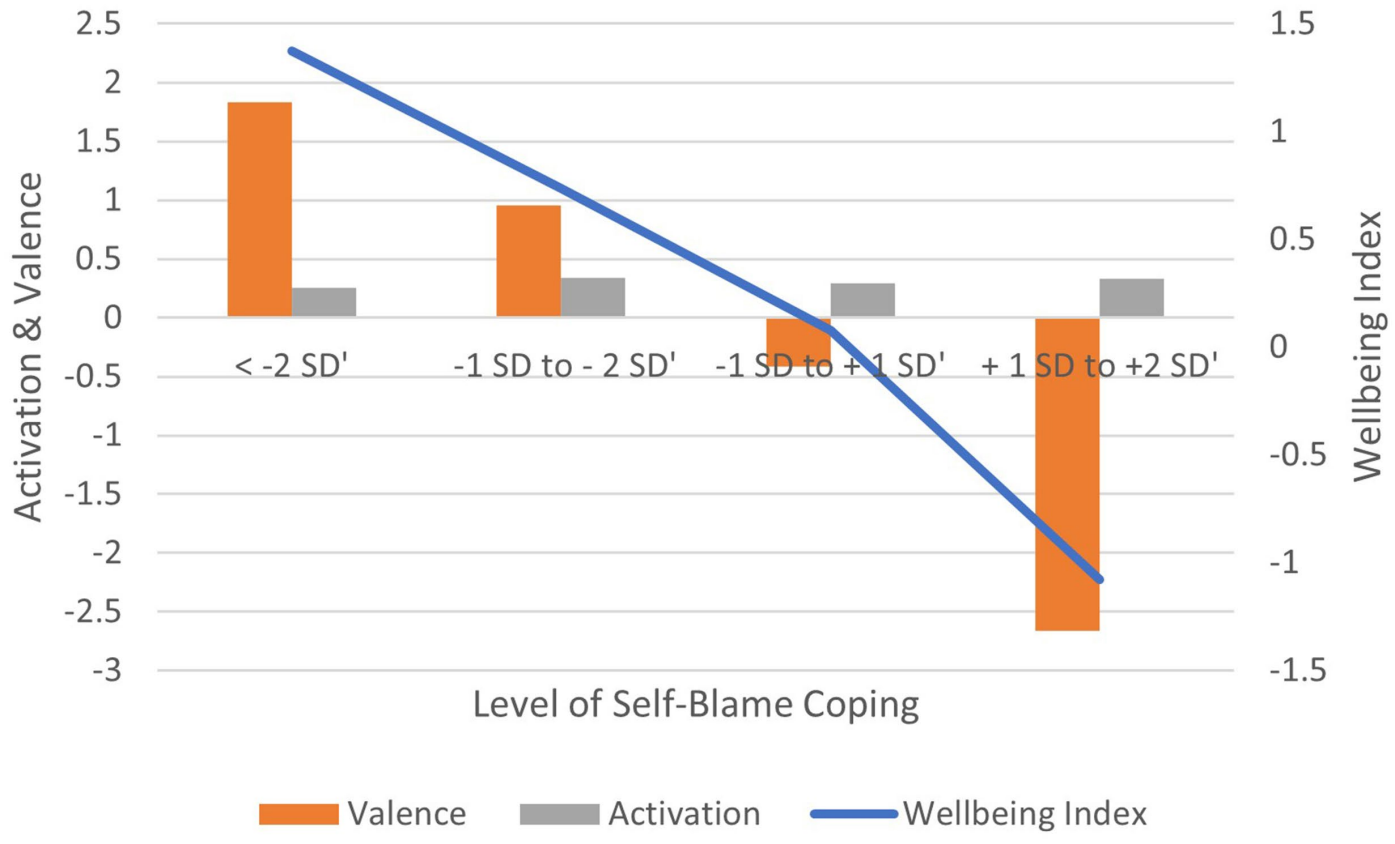
AgileBrain valence, activation, and wellbeing index scores by levels of self-blame coping.

Correlation between Self-Blame-based coping scores and: (1) activation is non-significant (*r* = 0.033, ns); (2) valence is negative and significant (*r* = −0.193, *p* < 0.01); (3) wellbeing index is negative and significant (*r* = −0.184, *p* < 0.01) (Figure 10). In terms of effect size for the wellbeing index, Cohen’s *d* = 2.69, an extremely large effect.

Correlation between Acceptance-based coping scores and: (1) activation is non-significant (*r* = 0.022, ns); (2) valence is non-significant (*r* = −0.004, ns); (3) wellbeing index is non-significant (*r* = −0.035, ns) (Supplementary Figure S22). In terms of effect size for the wellbeing index, Cohen’s *d* = 2.78, an extremely large effect.

**Figure 11:**
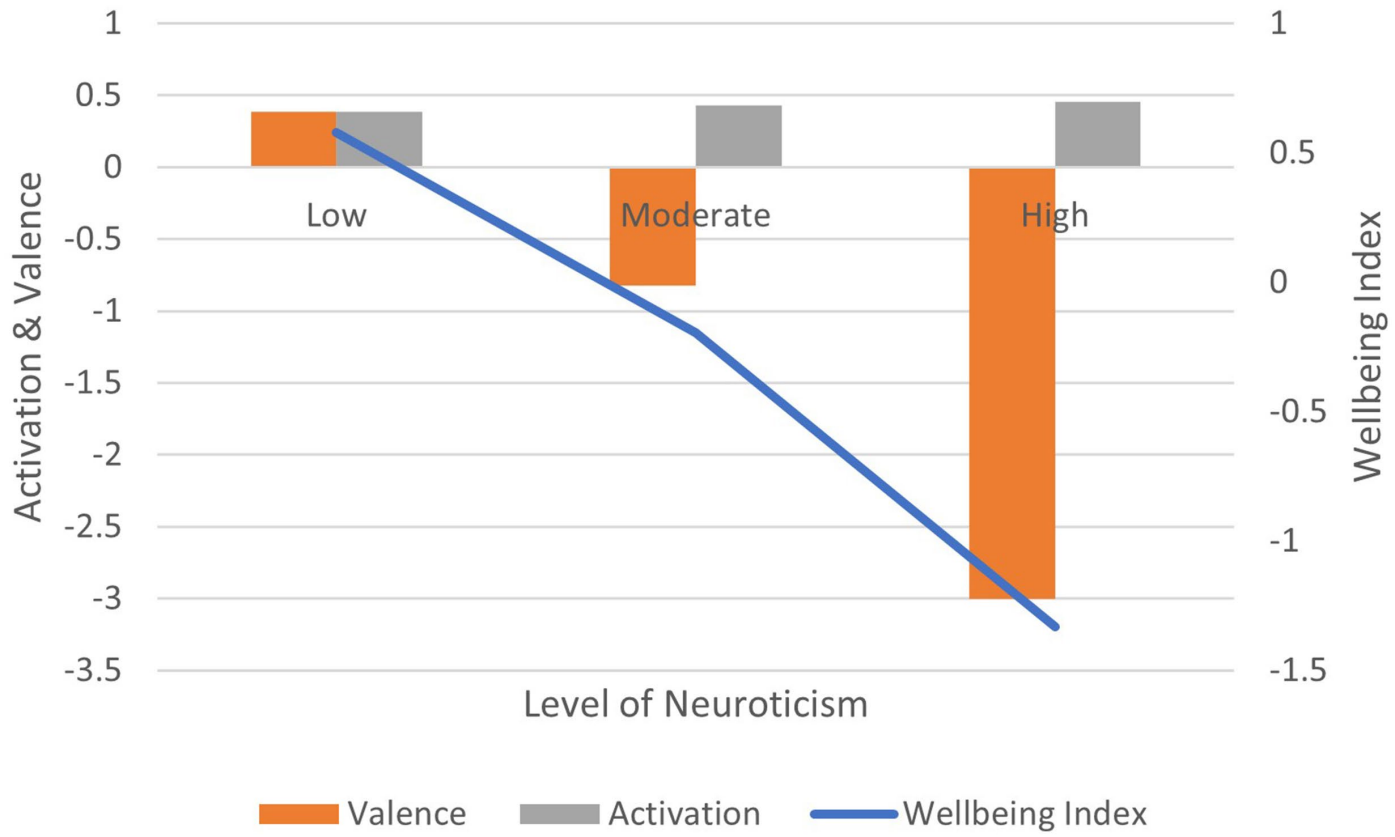
AgileBrain valence, activation, and wellbeing index scores by BFI-15 levels of neuroticism.

Correlation between the self-rated neuroticism and: (1) activation is positive and significant (*r* = 0.138, *p* < 0.01); (2) valence is negative and significant (*r* = −0.172, *p* < 0.01); (3) wellbeing index is negative and significant (*r* = −0.175, *p* < 0.01) (Figure 11). In terms of effect size for the wellbeing index, Cohen’s *d* = 2.45, an extremely large effect.

**Figure 12:**
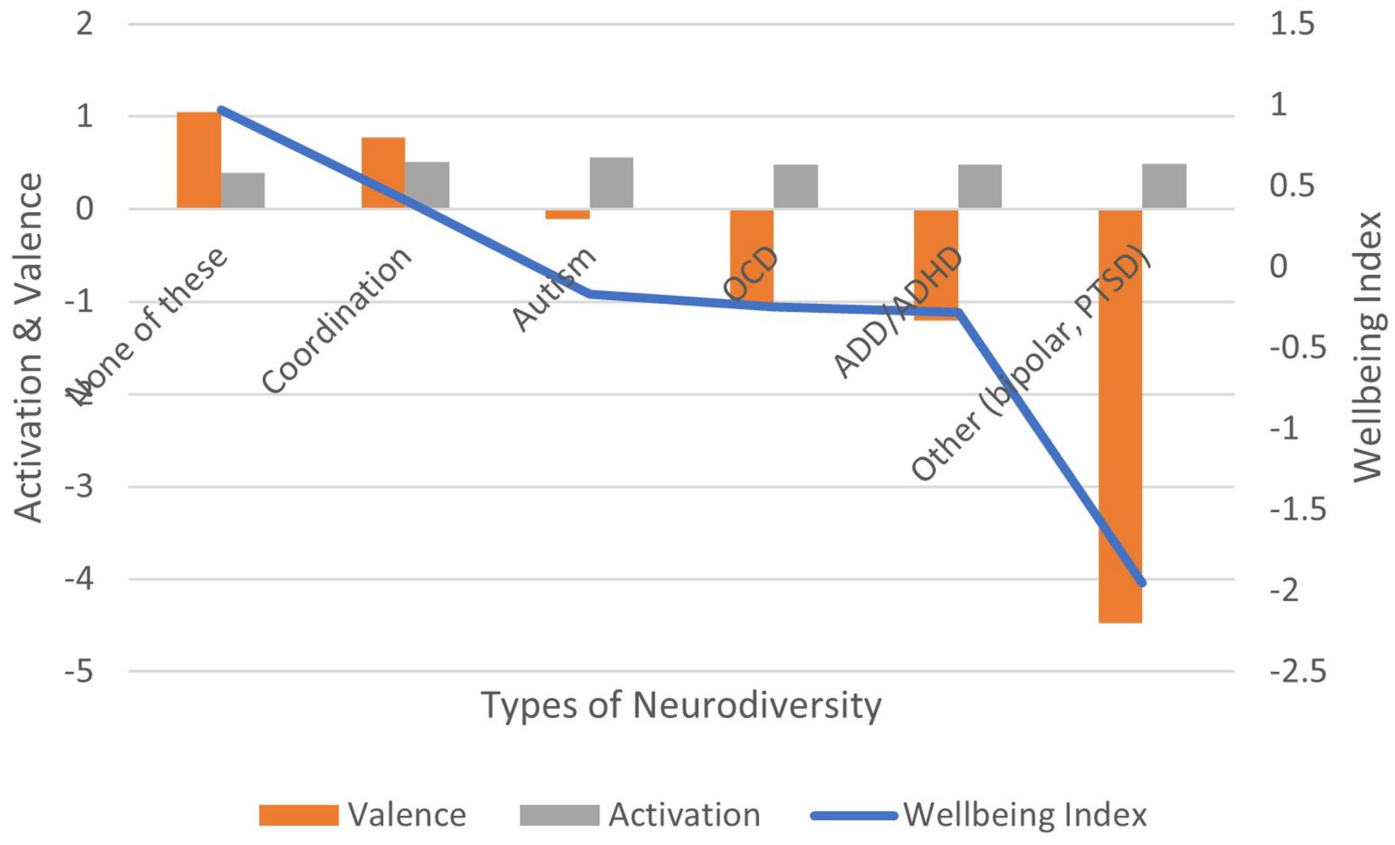
AgileBrain valence, activation, and wellbeing index scores by neurodiversity diagnoses.

Correlation between neurodiversity diagnosis (vs. no diagnosis) and: (1) activation is positive and significant (*r* = 0.150, *p* < 0.01); (2) valence is non-significant (*r* = −0.046, ns); (3) wellbeing index is non-significant (*r* = −0.036, ns) (Figure 12). In terms of effect size for the wellbeing index, Cohen’s *d* = 3.86, an extremely large effect.

**Figure 13:**
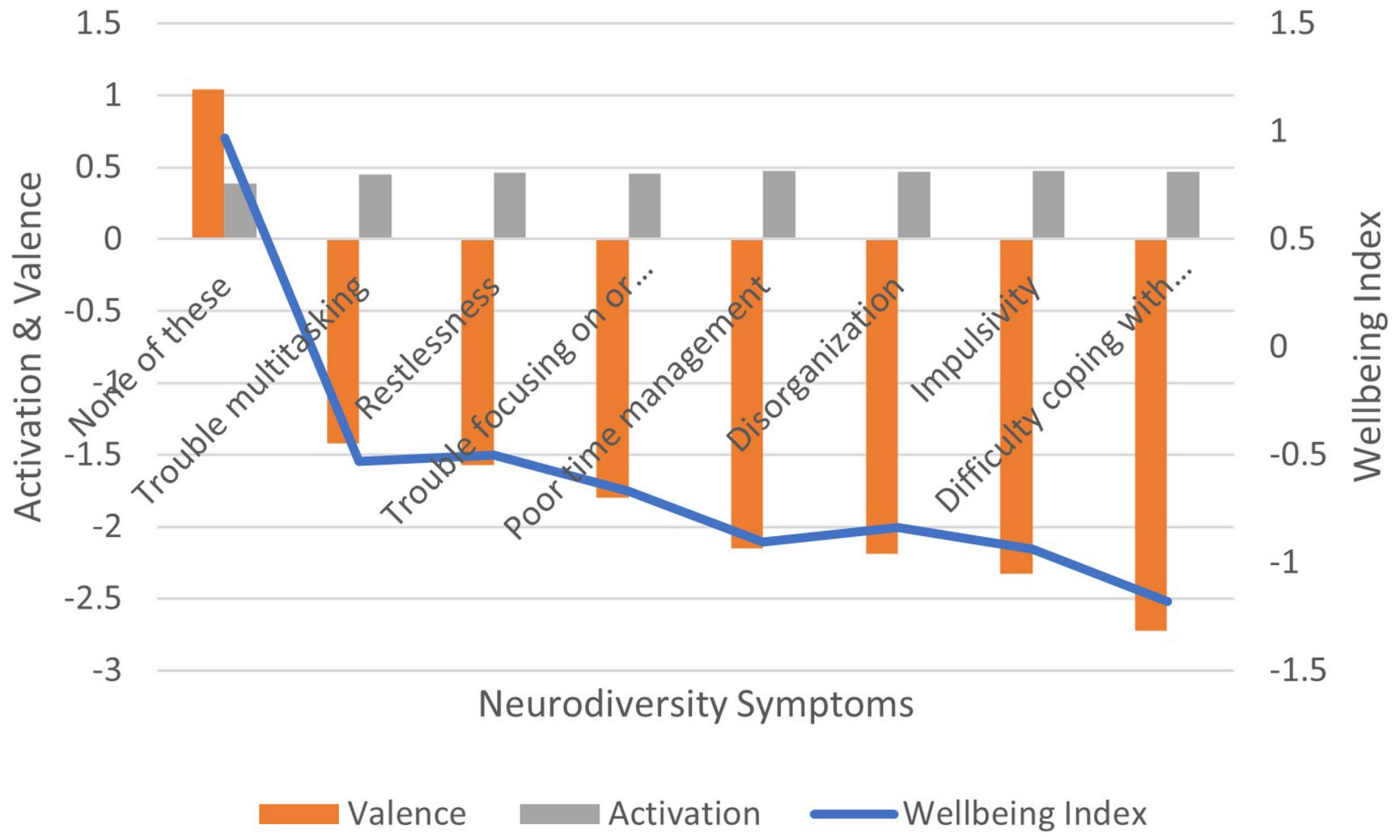
AgileBrain valence, activation, and wellbeing index scores by neurodiversity symptoms.

Correlation between the presence and absence of symptoms and: (1) activation is positive and significant (*r* = 0.108, *p* < 0.01); (2) valence is negative and significant (*r* = −0.144, *p* < 0.01); (3) wellbeing index is negative and significant (*r* = −0.111, *p* < 0.01) (Figure 13). In terms of effect size for the wellbeing index, Cohen’s *d* = 3.46, an extremely large effect.

**Figure 14:**
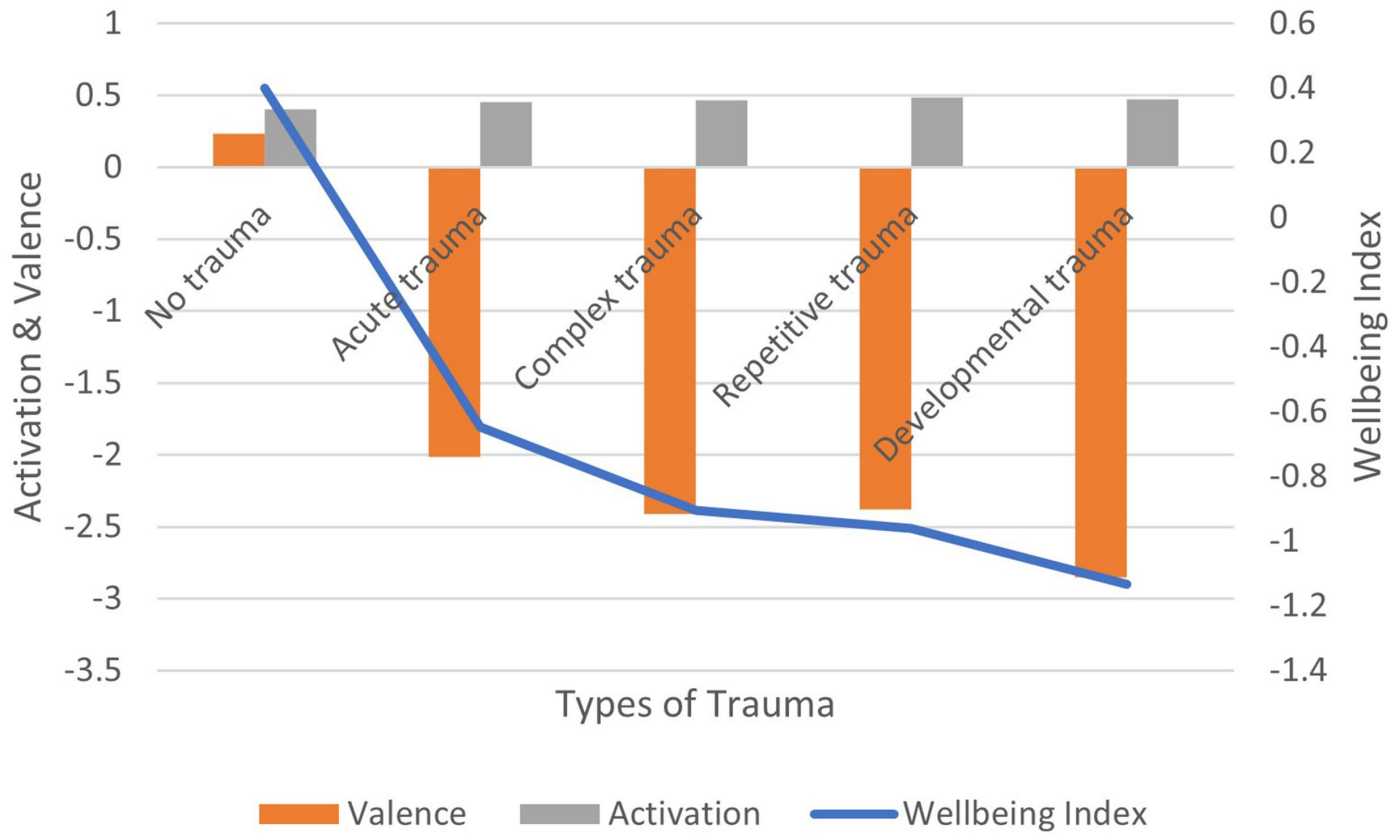
AgileBrain valence, activation, and wellbeing index scores by levels of trauma.

Correlation between the experience of trauma (vs. no trauma) and: (1) activation is positive and significant (*r* = 0.108, *p* < 0.01); (2) valence is negative and significant (*r* = −0.135, *p* < 0.01); (3) wellbeing index is negative and significant (*r* = −0.122, *p* < 0.01) (Figure 14). In terms of effect size for the wellbeing index, Cohen’s *d* = 2.83, an extremely large effect.

### 3.3 Discriminant function analysis

The value of a screening tool is derived largely from its ability to differentially identify individuals according to their level of wellbeing. Accordingly, discriminant function analysis (using standardized Fisher coefficients) was conducted to evaluate the ability of AgileBrain scores to distinguish between groups at different levels of wellbeing according to the standard levels suggested by the authors of the PHQ-9 and GAD-7. These analyses were conducted at three different cut-points for levels of anxiety and depression:

1. Highest vs. lowest level
2. Highest two levels vs. lowest two levels
3. Splitting the full scale at the median level into an upper and lower group

Classification results were analyzed for all cases in the analysis as well for cross-validated models classified by the functions derived from all cases other than the predicted case (i.e., holdouts); cross-validation is generally suggested to avoid model over-fitting. Results of these analyses show that as an indirect assessment, AgileBrain is very effective in differentiating those at high vs. low levels of emotional wellbeing. Using all cases, high vs. low wellbeing individuals are accurately classified at rates from a low of 69.2% to a high of 92.2%. When cross-validated using holdout cases, classification accuracy ranges from a low of 64.0% accuracy to a high of 76.7% (Supplementary Table S1).

## 4 Discussion

### 4.1 Summary of findings

AgileBrain scores are consistently correlated with indicators of compromised emotional wellbeing. When used as a screening tool, discriminant function analysis revealed that high vs. low depression is accurately detected at rates from 75.4% accuracy to 92.2% accuracy. High vs. low anxiety is accurately detected at rates from 76.7% accuracy to 81.2% accuracy. Every effect size calculated in the present study is extremely large, indicating minimal overlap in wellbeing index scores between populations who experience high levels of each threat to wellbeing and those who do not; Cohen’s *d* values range from 1.52 to 3.86, suggesting that AgileBrain is extremely efficient in differentiating levels of wellbeing.

In nearly every case, the severity of the condition is positively correlated with (1) emotional activation and negatively correlated with (2) valence and (3) wellbeing index. The evidence suggests that AgileBrain consistently differentiates between a variety of threats to emotional wellbeing including depression, anxiety, loneliness, neuroticism, neurodiversity diagnosis and symptoms, and traumatic experiences. Only one of the comparison measures failed to show a significant correlation with one of the three AgileBrain summary metrics, the correlation between stress level and activation (Supplementary Table S2). This finding is non-problematic to the extent that scholars in the stress field have demonstrated that not all stress is bad or harmful, rather there can be good stress and bad stress (17).

The pattern of results among the various strategies used to cope with stress is revealing (i.e., for those scoring between one and two standard deviations above the mean for each strategy). There is consensus that social support and spirituality-based coping strategies are particularly adaptive, and we find positive wellbeing index scores for those using these strategies. Distraction as a coping strategy is viewed as effective on a short-term basis, and we find a smaller average positive wellbeing index score for those using this approach. At the other end of the spectrum, we see strongly negative average wellbeing index scores for those relying on self-blame, avoidance, and externalization. Intriguingly, we also find a strongly negative average wellbeing index associated with the “adaptive” strategy of acceptance; this may be related to the increased level of harmful stress associated with situations that necessitate acceptance (Supplementary Table S3).

### 4.2 Implications

These results suggest that AgileBrain shows construct validity as a screening tool for detecting compromised wellbeing in the general population. AgileBrain scores demonstrated robust discrimination of wellbeing levels in four large general population samples. This, coupled with user preference for brief, gamified digital assessments (18, 19) makes AgileBrain a viable candidate for evaluating population wellbeing in large populations.

### 4.3 Strengths and limitations

A strength of the current study is derived from the use of large population-representative samples of both sufferers and non-sufferers of threats to emotional wellbeing, providing an illustration of its potential as a population screening tool. The major limitation of this study is that the relationships between measures represent point-in-time “snapshots” rather than longitudinal measures. Longitudinal research is currently underway to investigate AgileBrain’s sensitivity in detecting change in wellbeing over time within clinical settings.

## 5 Conclusion

In this exploratory analysis, AgileBrain demonstrated construct validity and sensitivity to differences in a series of different clinical outcome measures in two distinct, large, population-representative samples. These analyses support the use of AgileBrain as tool for indirectly screening large populations for compromised wellbeing. Given widespread preference for gamified digital experiences and the statistical benefits of measures that are normally distributed, AgileBrain could serve as an alternative to intrusive direct questioning about emotional wellbeing in the monitoring and evaluation of large populations.

## Data Availability

The raw data supporting the conclusions of this article will be made available by the authors, without undue reservation.

## Footnotes

^1^The November 2021 sample is representative of the full-time working population aged 25 to 54.

## References

[1] Pincus JD. The structure of human motivation. BMC Psychol. (2023b) 11:308. doi: 10.1186/s40359-023-01346-5 37798750 PMC10557177

[2] Rudrauf D David O Lachaux JP Kovach CK Martinerie J Renault B. Rapid interactions between the ventral visual stream and emotion-related structures rely on a two-pathway architecture. J Neurosci. (2008) 28:2793–803. doi: 10.1523/JNEUROSCI.3476-07.2008, PMID: 18337409 PMC6670659

[3] Rudrauf D Lachaux JP Damasio A Baillet S Hugueville L Martinerie J. Enter feelings: somatosensory responses following early stages of visual induction of emotion. Int J Psychophysiol. (2009) 72:13–23. doi: 10.1016/j.ijpsycho.2008.03.015, PMID: 18938199

[4] Vahratian A Blumberg SJ Terlizzi EP Schiller JS. Symptoms of anxiety or depressive disorder and use of mental health care among adults during the COVID-19 pandemic — United States, august 2020–February 2021. MMWR Morb Mortal Wkly Rep. (2021) 70:490–4. doi: 10.15585/mmwr.mm7013e2, PMID: 33793459 PMC8022876

[5] Pincus JD. Personal communications with mental health clinicians in the SUNY system (Mohawk Valley) and US military Fort Liberty. Boston, MA, USA (2024).

[6] Pincus JD. Wellbeing as human motivation: implications for theory, methods, and practice. Integr Psychol Behav Sci. (2023a) 10.1007/s12124-022-09737-w PMC9797252 36577907

[7] Pincus JD. Theoretical and empirical foundations for a unified pyramid of human motivation. Integr Psychol Behav Sci. (2024) 58, 731–756. doi: 10.1007/s12124-022-09700-9 35595972 PMC11052772

[8] U.S. Bureau of Labor Statistics. (2023). Labor force statistics from the current population survey

[9] Kroenke K Spitzer RL Williams JB. The PHQ-9: validity of a brief depression severity measure. J Gen Intern Med. (2001) 16:606–13. doi: 10.1046/j.1525-1497.2001.016009606.x 11556941 PMC1495268

[10] Eaton WW Muntaner C Smith C Tien A Ybarra M. Center for epidemiologic studies depression scale: review and revision In: ME Maruish, editor. The use of psychological testing for treatment planning and outcomes assessment. New York: Lawrence Erlbaum Associates Publishers (2004). 363–77.

[11] Herniman SE Allott KA Killackey E Hester R Cotton SM. The psychometric validity of the Center for Epidemiological Studies–Depression Scale (CES-D) in first episode schizophrenia spectrum. Psychiatry Res. (2017) 252:16–22. doi: 10.1016/j.psychres.2017.02.023, PMID: 28237759

[12] Spitzer RL Kroenke K Williams JB Löwe B. A brief measure for assessing generalized anxiety disorder: the GAD-7. Arch Intern Med. (2006) 166:1092–7. doi: 10.1001/archinte.166.10.1092, PMID: 16717171

[13] Hughes ME Waite LJ Hawkley LC Cacioppo JT. A short scale for measuring loneliness in large surveys: results from two population-based studies. Res Aging. (2004) 26:655–72. doi: 10.1177/0164027504268574, PMID: 18504506 PMC2394670

[14] Cohen S Kamarck T Mermelstein R. Perceived stress scale. APA PsycTests. (1983). doi: 10.1037/t02889-000 6668417

[15] Carver CS. You want to measure coping but your protocol’s too long: consider the brief. Int J Behav Med. (1997) 4:92–100. doi: 10.1207/s15327558ijbm0401_6, PMID: 16250744

[16] Lang FR John D Ludtke O Schupp J Wagner GG. Short assessment of the big five: robust across survey methods except telephone interviewing. Behav Res Methods. (2011) 43:548–67. doi: 10.3758/s13428-011-0066-z, PMID: 21424189 PMC3098347

[17] Herman JP Maroun M Richter-Levin G. Good stress, bad stress and very bad stress. Stress. (2015) 18:267–8. doi: 10.3109/10253890.2015.1087091, PMID: 26383031

[18] Salzberg CA Kahn CN III Foster NE Demehin AA Guinan ME Ramsey P. Modernizing the HCAHPS survey. Haifa, Israel: Recommendations from patient experience leaders American Hospital Association (2019).

[19] Zhu S Guo Q Yang HH. Beyond the traditional: a systematic review of digital game-based assessment for students’ knowledge, skills, and affections. Sustain For. (2023) 15:4693. doi: 10.3390/su15054693

[20] American Psychiatric Association. (2022). Diagnostic and statistical manual of mental disorders (5th ed.). Washington, DC, USA: American Psychiatric Association Publishing.

[21] Pilch I Wardawy P Probierz E. The predictors of adaptive and maladaptive coping behavior during the COVID-19 pandemic: the protection motivation theory and the big five personality traits. PLoS One. (2021). doi: 10.1371/journal.pone.0258606 PMC8525766 34665837

