# Supplementary figures and images for: Validation of a brief image elicitation task as an indicator of subjective wellbeing in the general population
Source: Front Public Health. 2024 Oct 22;12:1435144. doi: 10.3389/fpubh.2024.1435144 (PMC11534616; doi:10.3389/fpubh.2024.1435144)

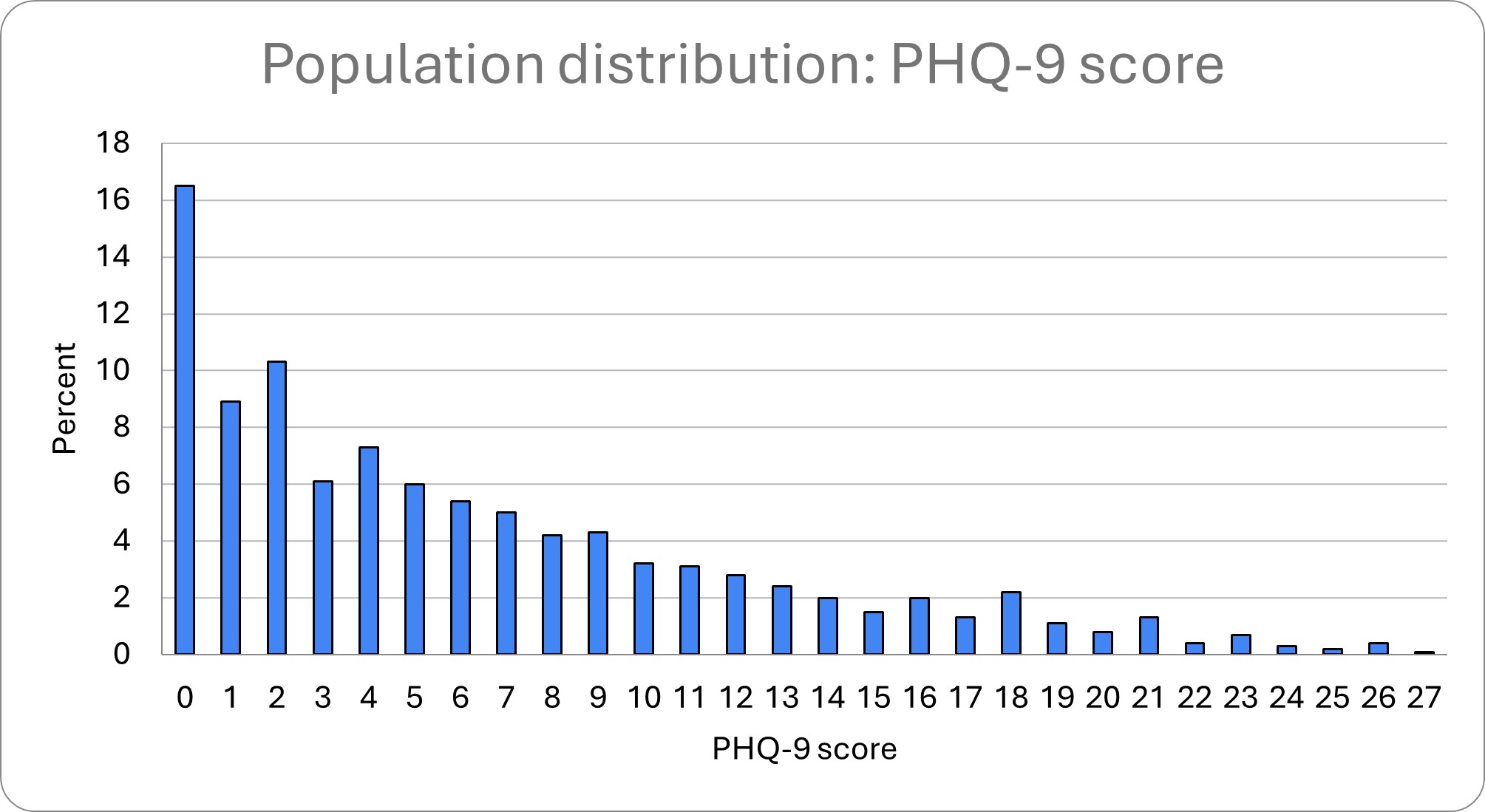

Supplement: Supplementary file 1 [file Data_Sheet_1.zip › Supplementary Figures/Figure 1 (SM). Population distribution of PHQ-9 scores.jpg]

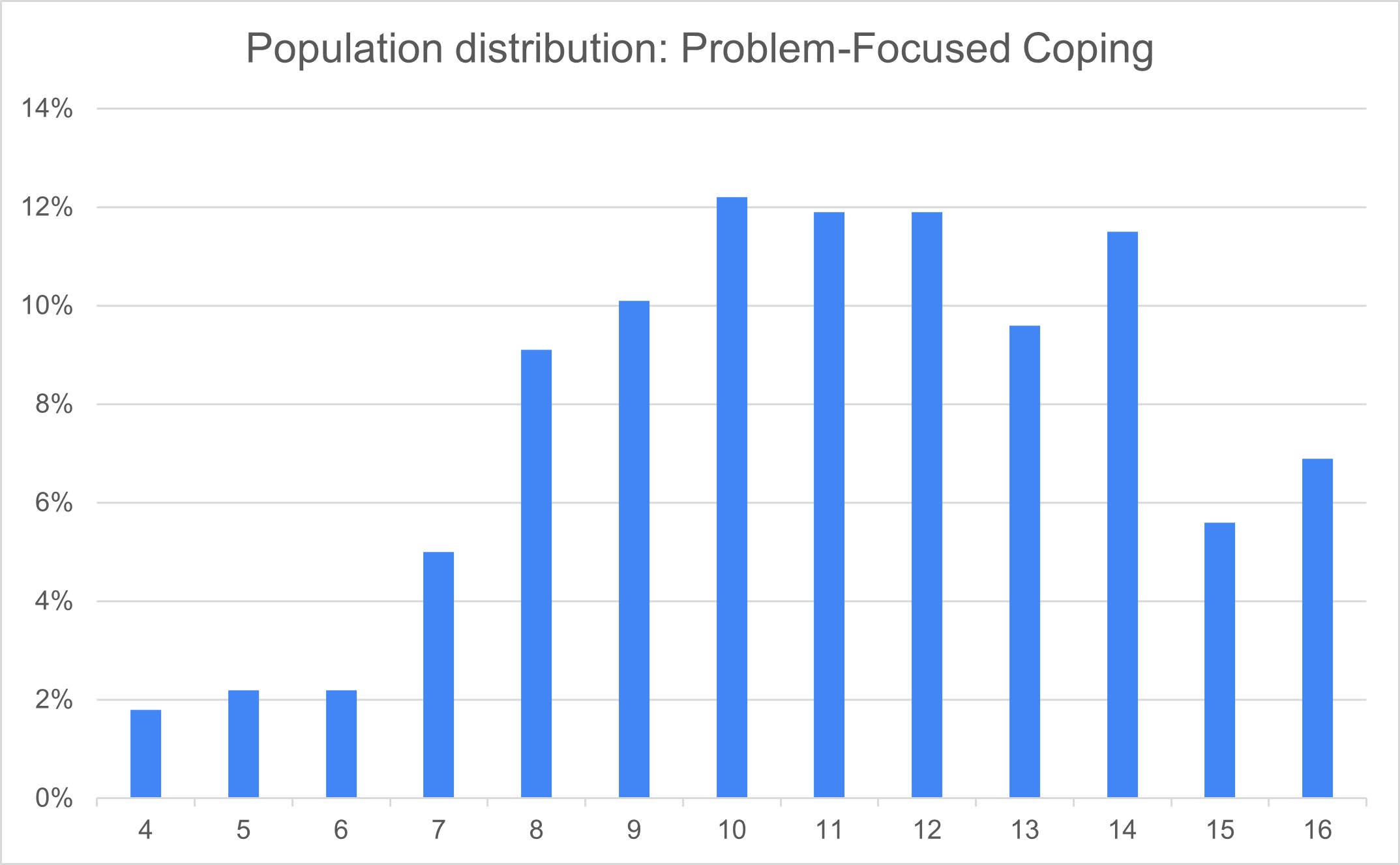

Supplement: Supplementary file 1 [file Data_Sheet_1.zip › Supplementary Figures/Figure 10 (SM). Population distribution of problem-focused coping style.jpg]

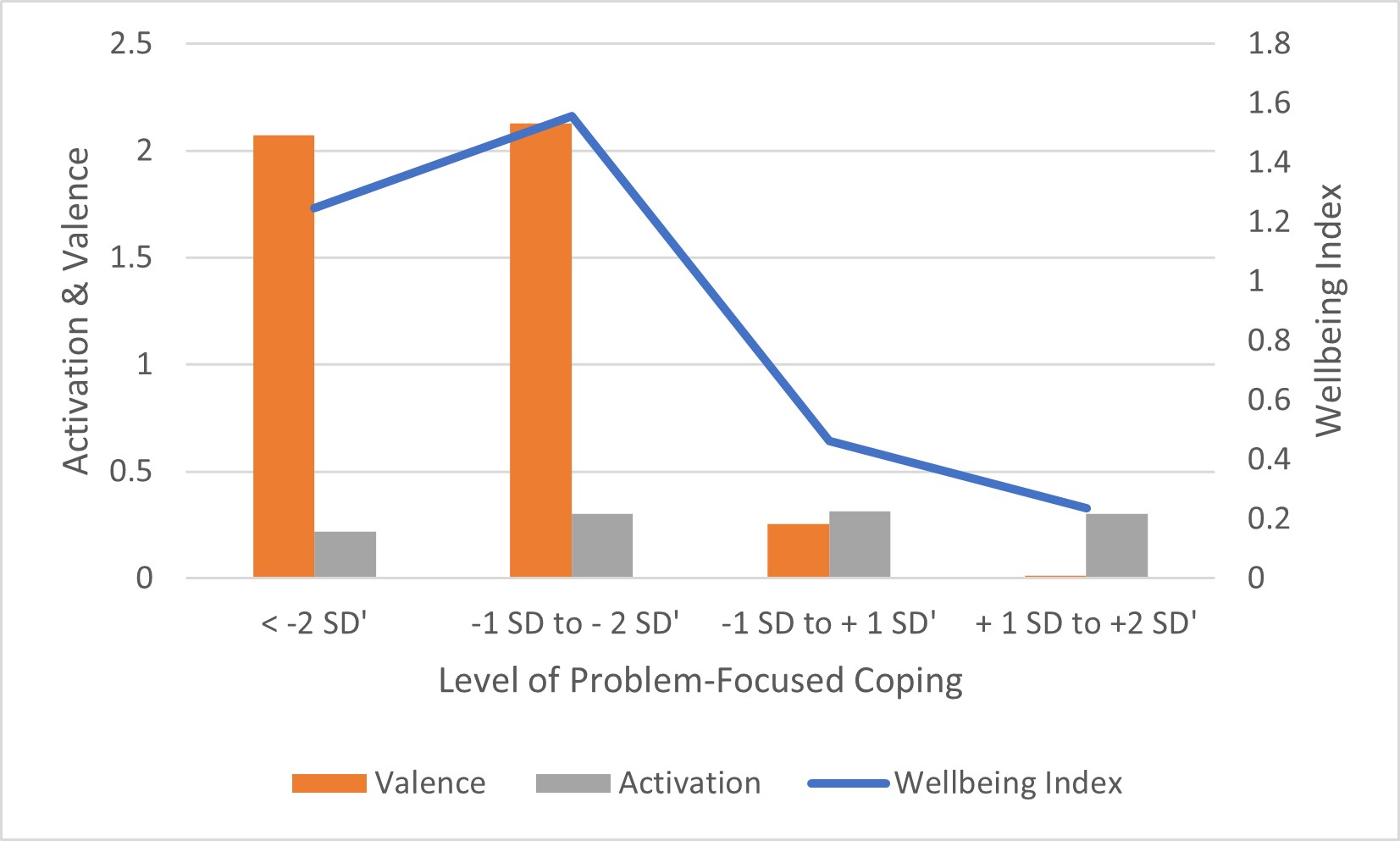

Supplement: Supplementary file 1 [file Data_Sheet_1.zip › Supplementary Figures/Figure 11 (SM). AgileBrain valence, activation, and wellbeing index scores by levels of problem-focused coping.jpg]

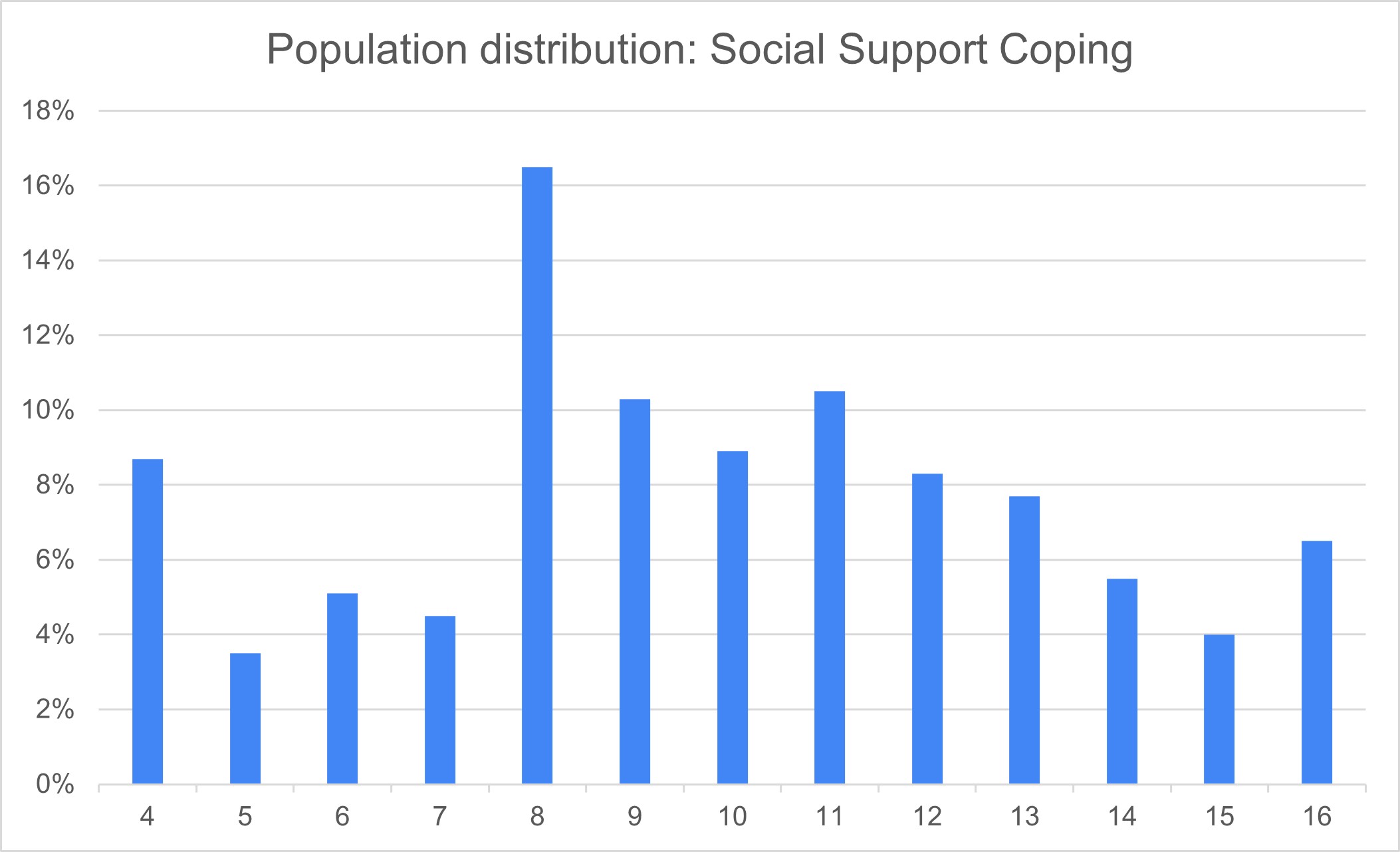

Supplement: Supplementary file 1 [file Data_Sheet_1.zip › Supplementary Figures/Figure 12 (SM). Population distribution of social support-based coping style.jpg]

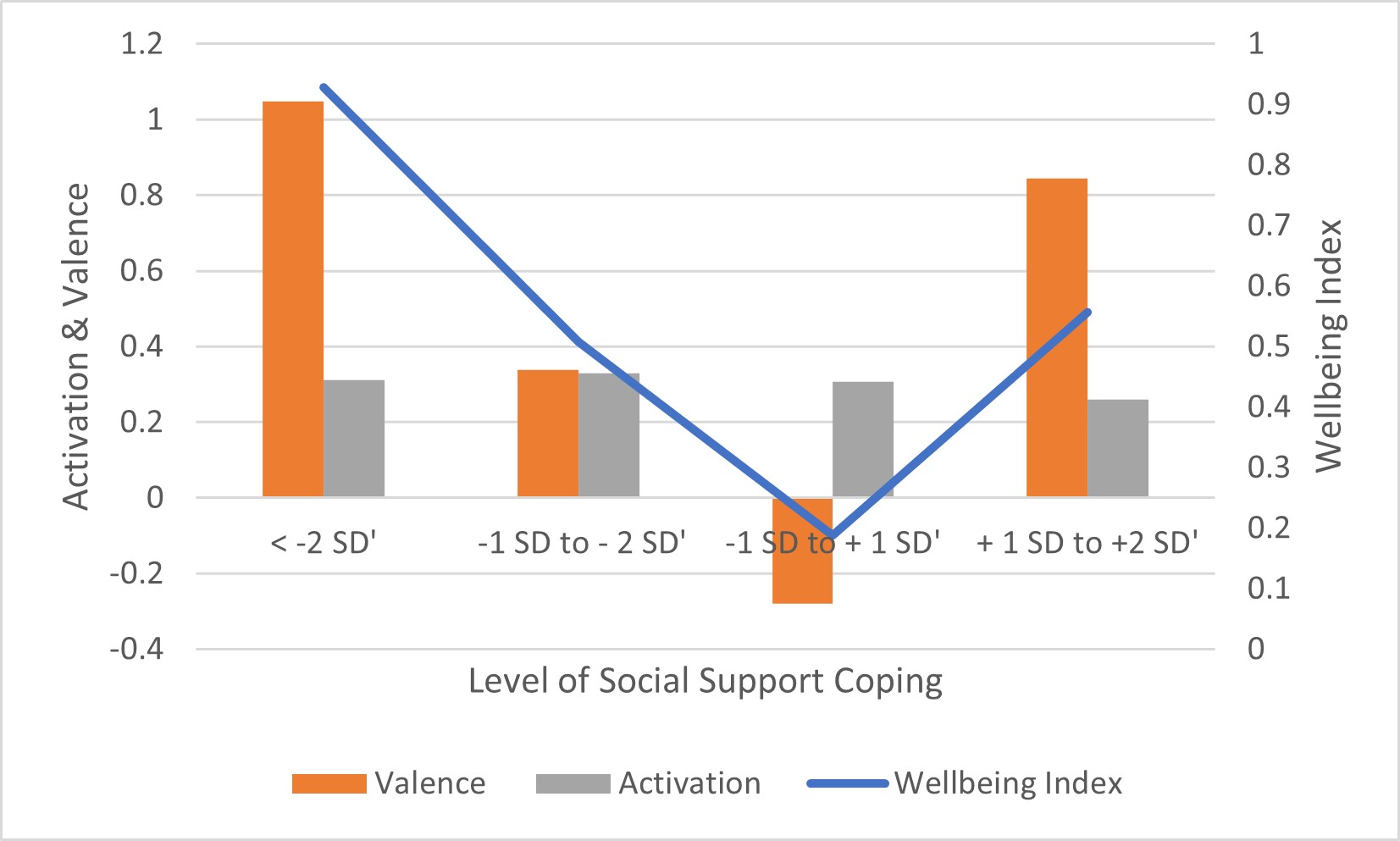

Supplement: Supplementary file 1 [file Data_Sheet_1.zip › Supplementary Figures/Figure 13 (SM). AgileBrain valence, activation, and wellbeing index scores by levels of social support-based coping.jpg]

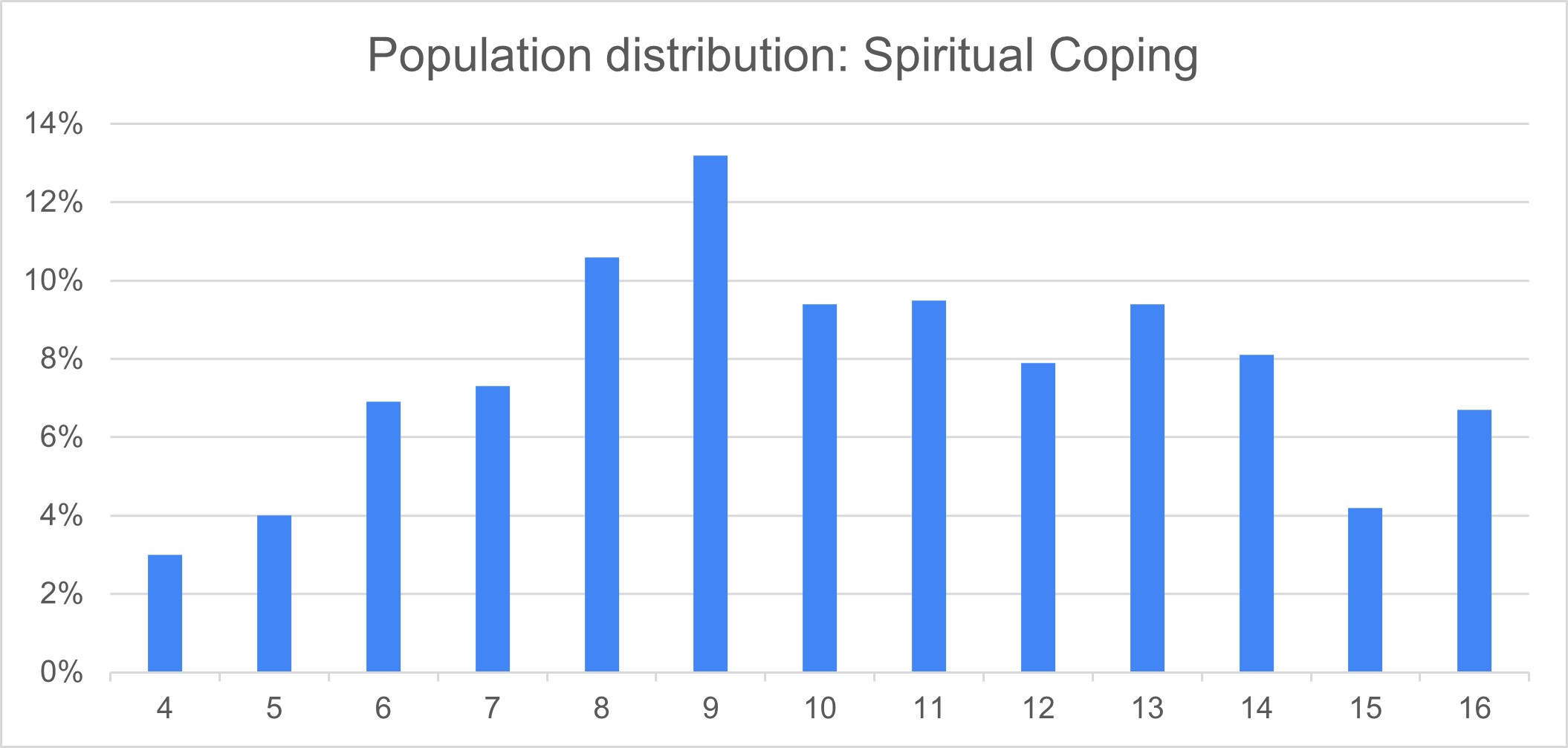

Supplement: Supplementary file 1 [file Data_Sheet_1.zip › Supplementary Figures/Figure 14 (SM). Population distribution of spiritual coping style.jpg]

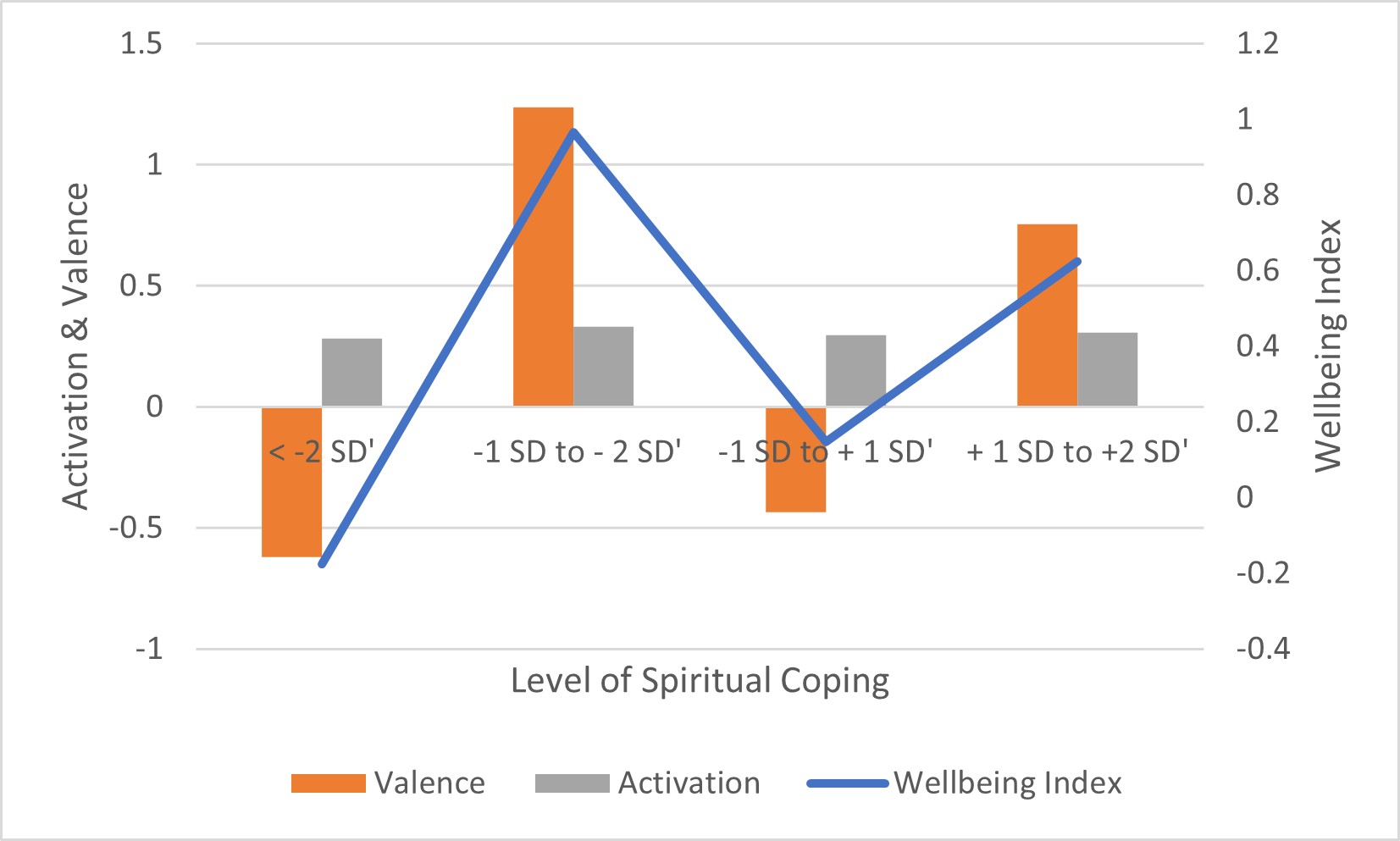

Supplement: Supplementary file 1 [file Data_Sheet_1.zip › Supplementary Figures/Figure 15 (SM). AgileBrain valence, activation, and wellbeing index scores by levels of spiritual coping.jpg]

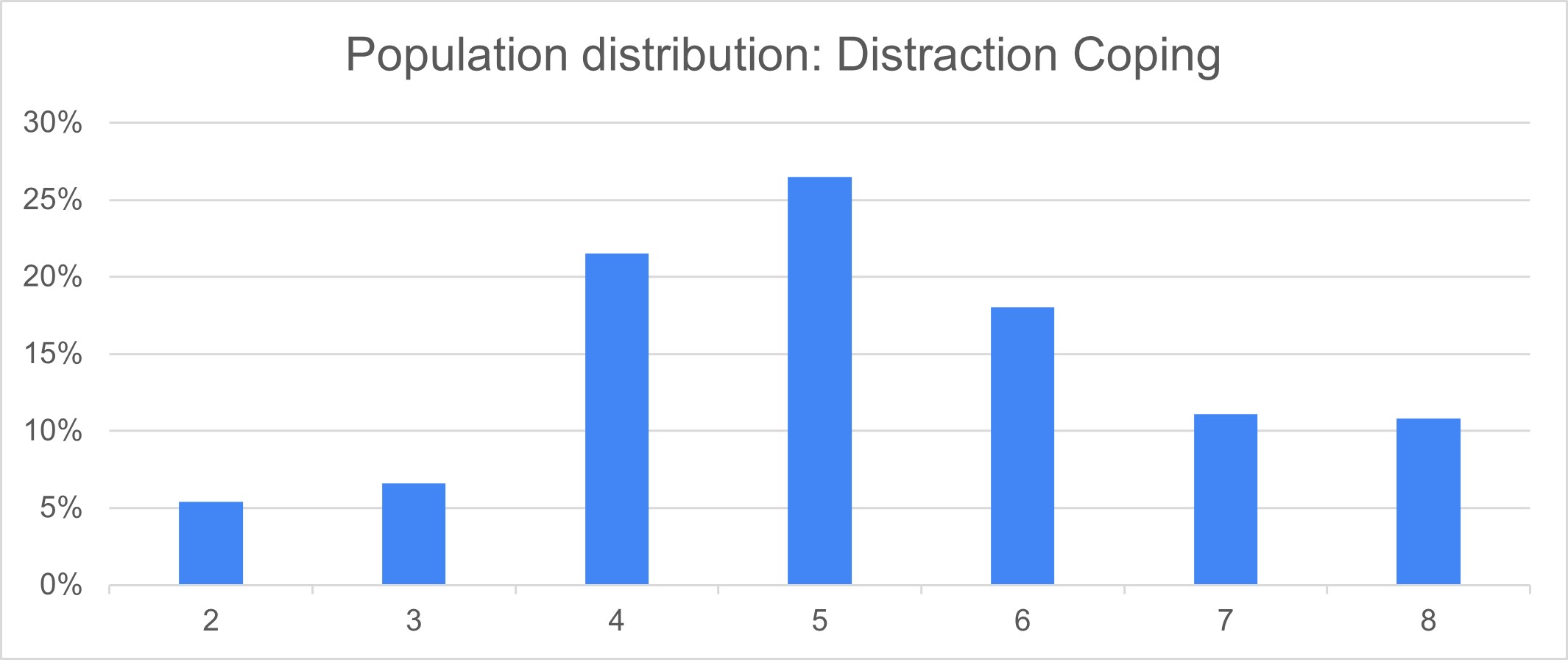

Supplement: Supplementary file 1 [file Data_Sheet_1.zip › Supplementary Figures/Figure 16 (SM). Population distribution of distraction-based coping style.jpg]

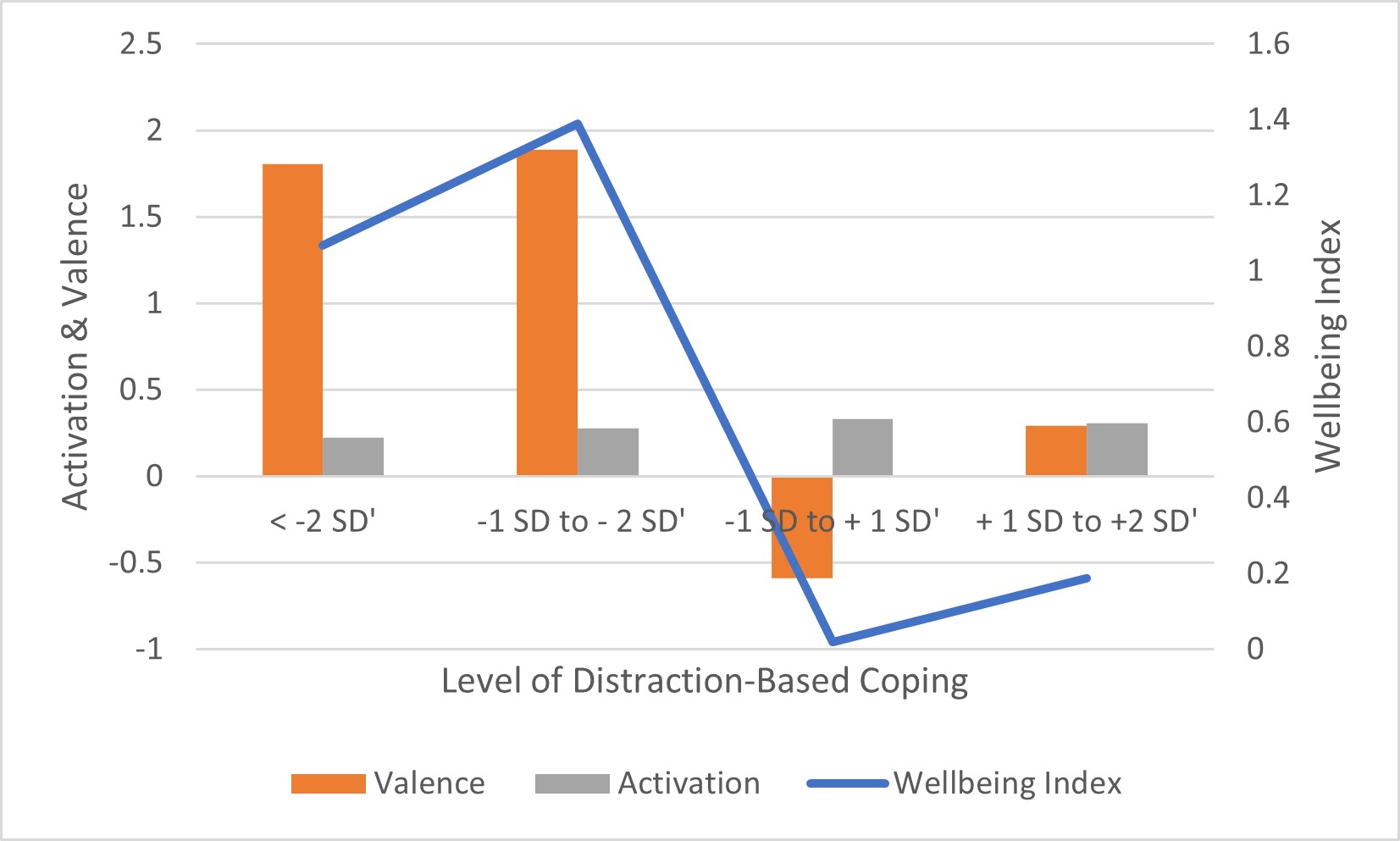

Supplement: Supplementary file 1 [file Data_Sheet_1.zip › Supplementary Figures/Figure 17 (SM). AgileBrain valence, activation, and wellbeing index scores by levels of distraction-based coping.jpg]

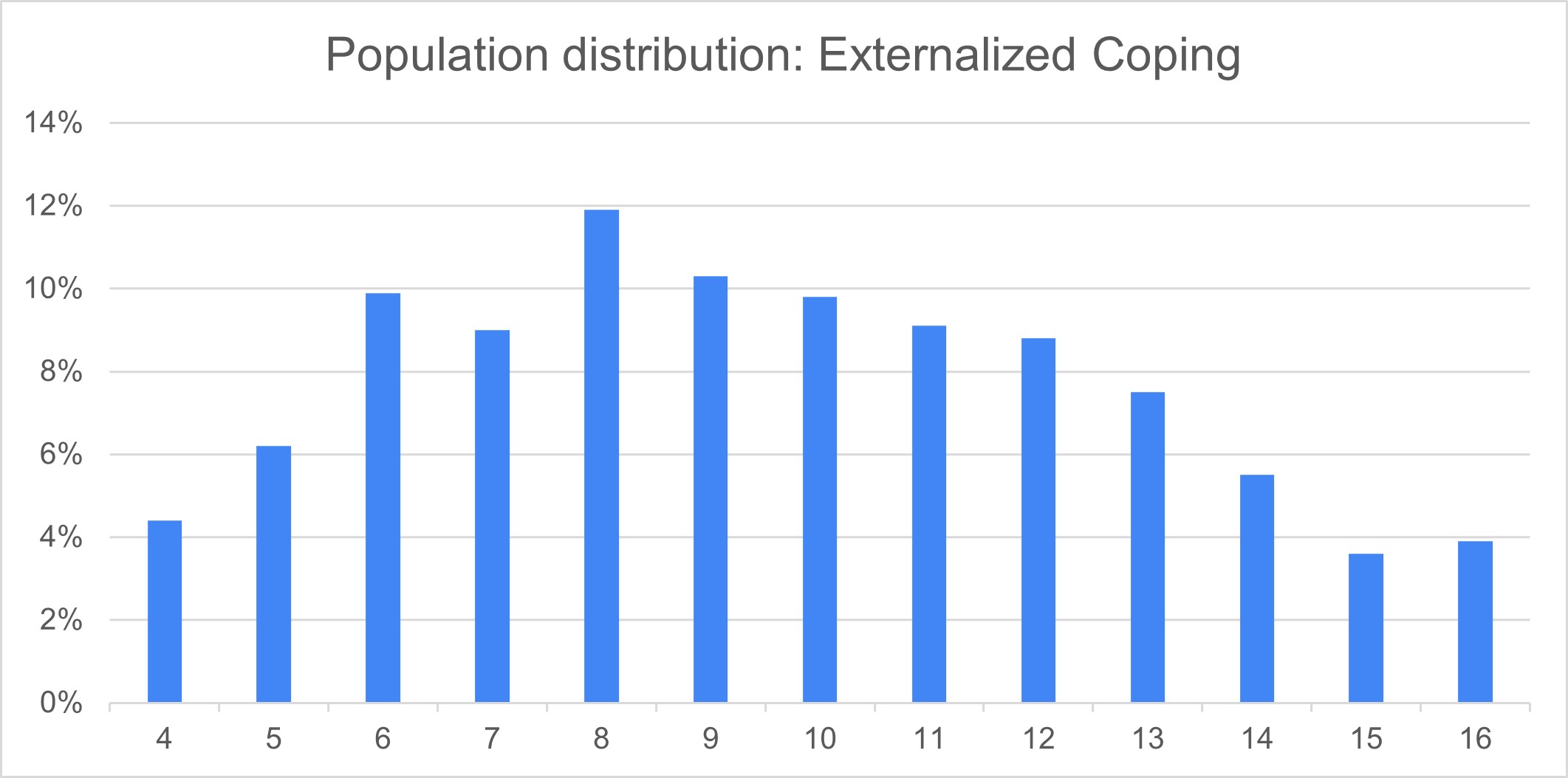

Supplement: Supplementary file 1 [file Data_Sheet_1.zip › Supplementary Figures/Figure 18 (SM). Population distribution of externalizing coping style.jpg]

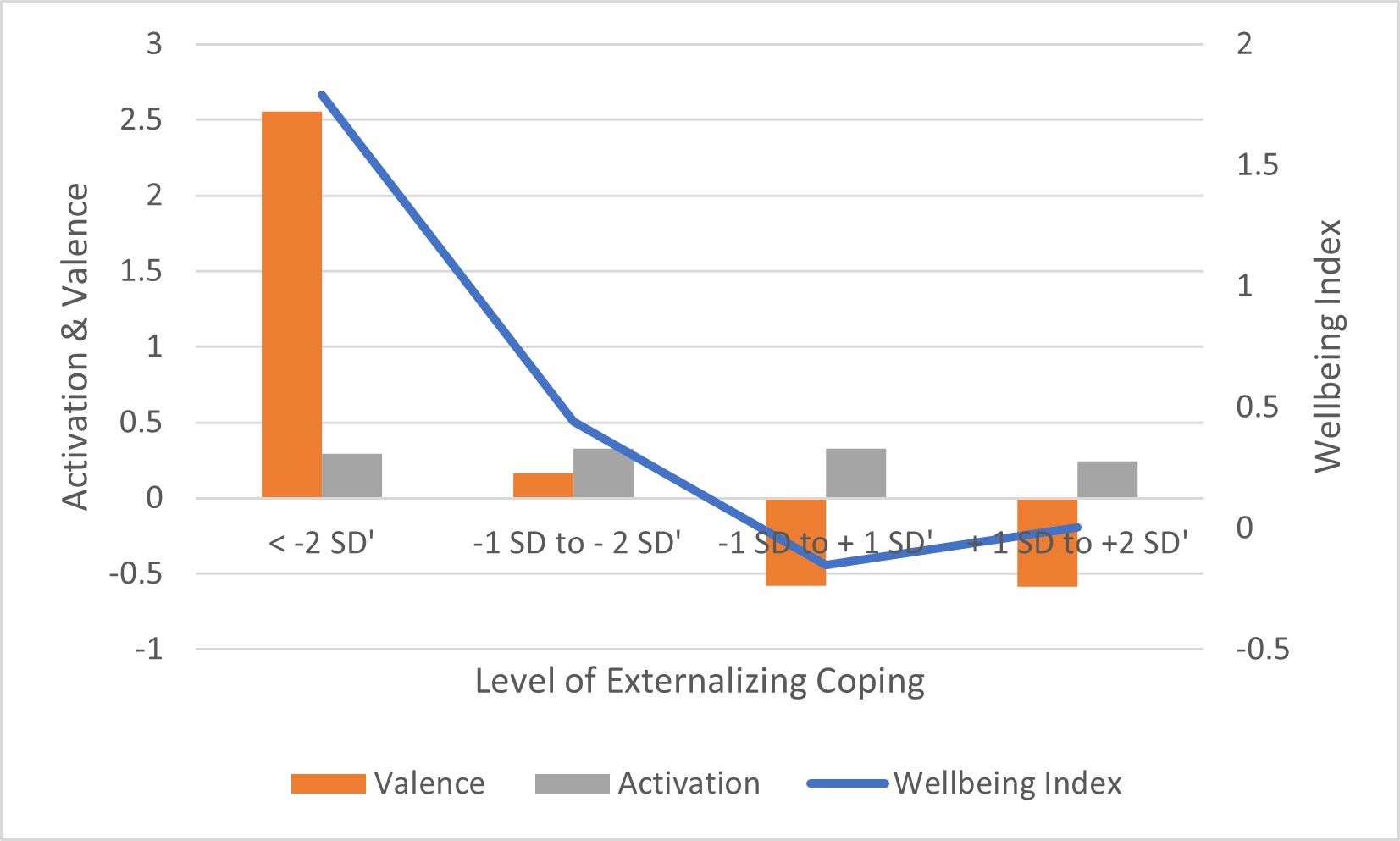

Supplement: Supplementary file 1 [file Data_Sheet_1.zip › Supplementary Figures/Figure 19 (SM). AgileBrain valence, activation, and wellbeing index scores by levels of externalizing coping.jpg]

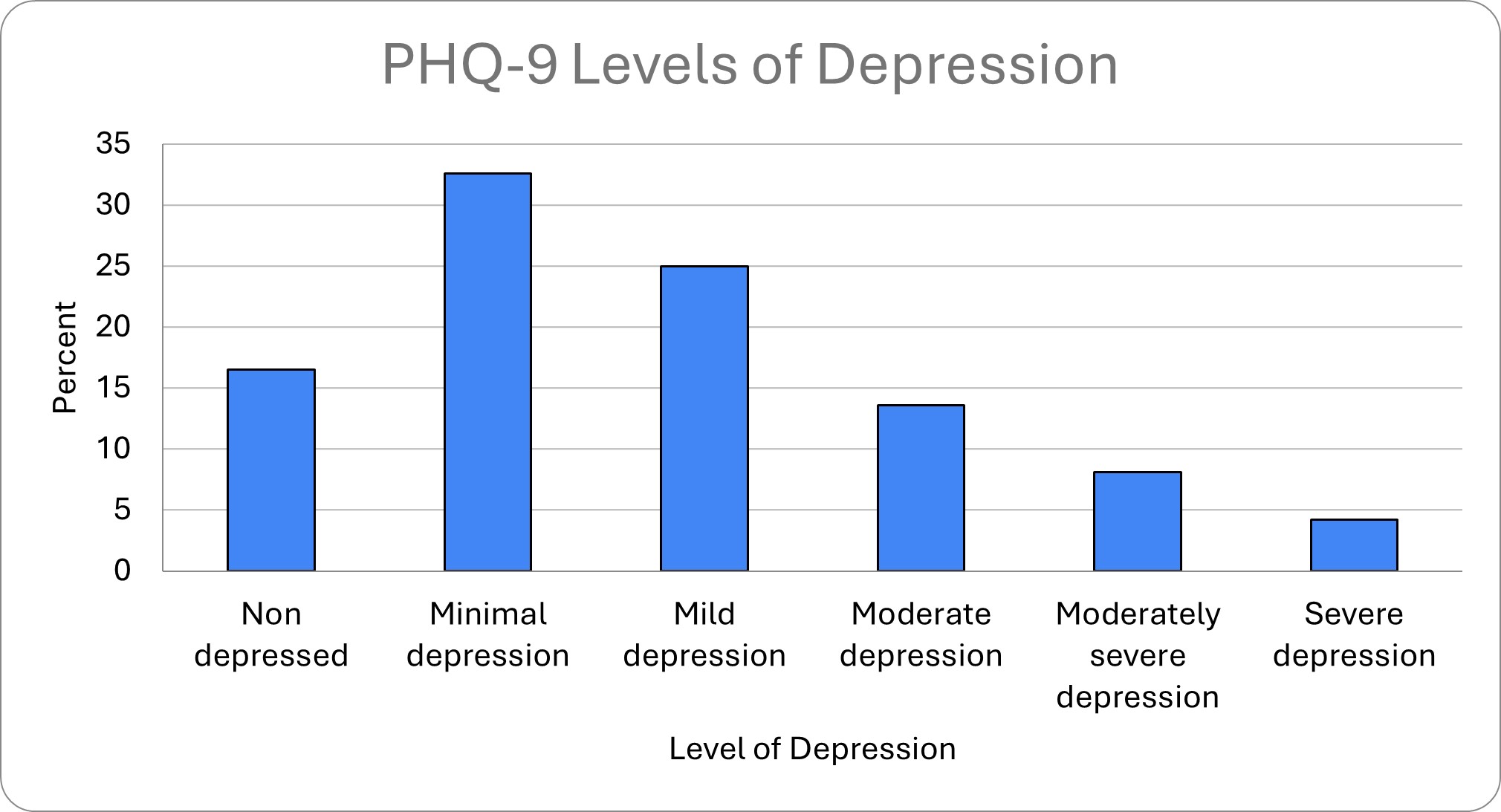

Supplement: Supplementary file 1 [file Data_Sheet_1.zip › Supplementary Figures/Figure 2 (SM). Population distribution of PHQ-9 standard levels of depression.jpg]

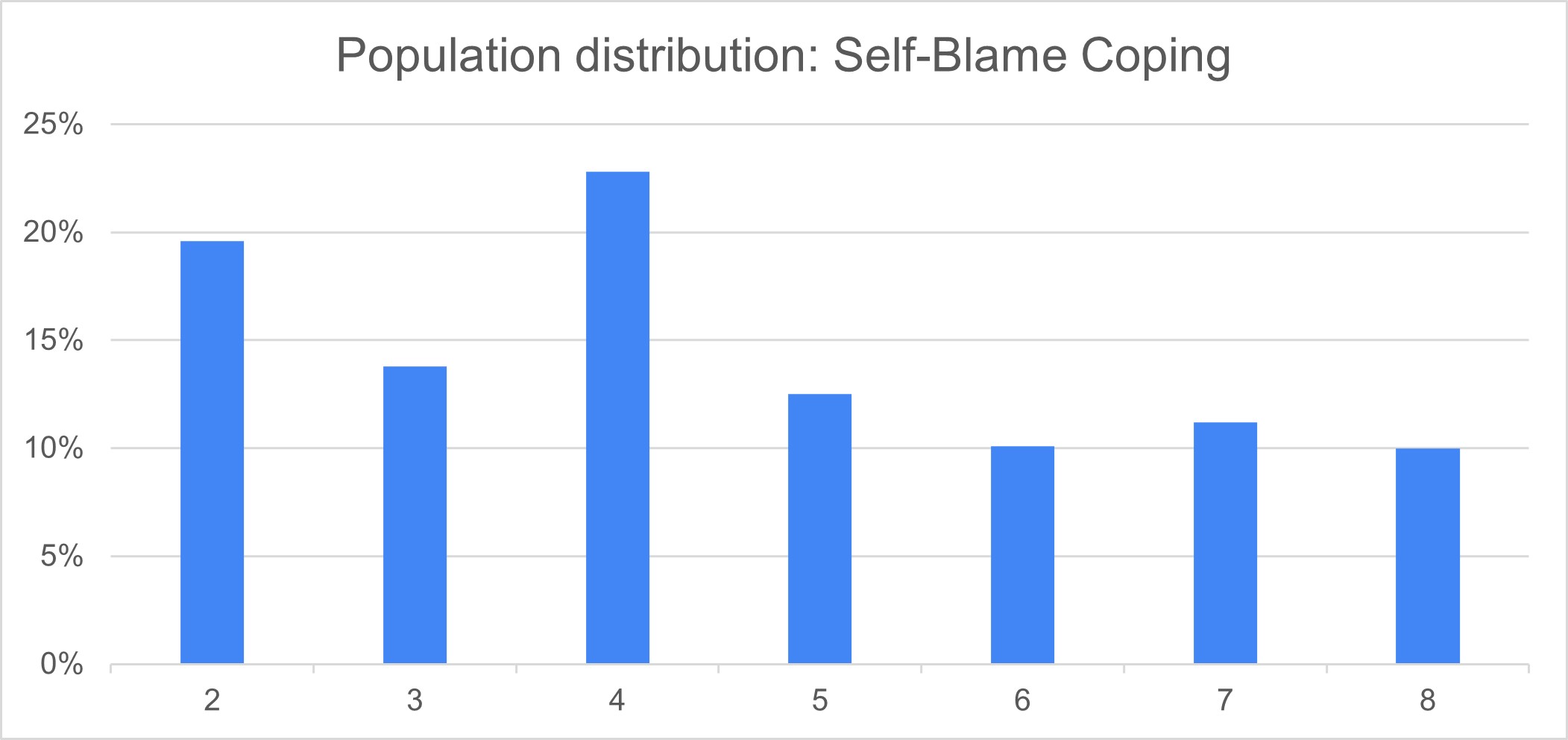

Supplement: Supplementary file 1 [file Data_Sheet_1.zip › Supplementary Figures/Figure 20 (SM). Population distribution of self-blame coping style.jpg]

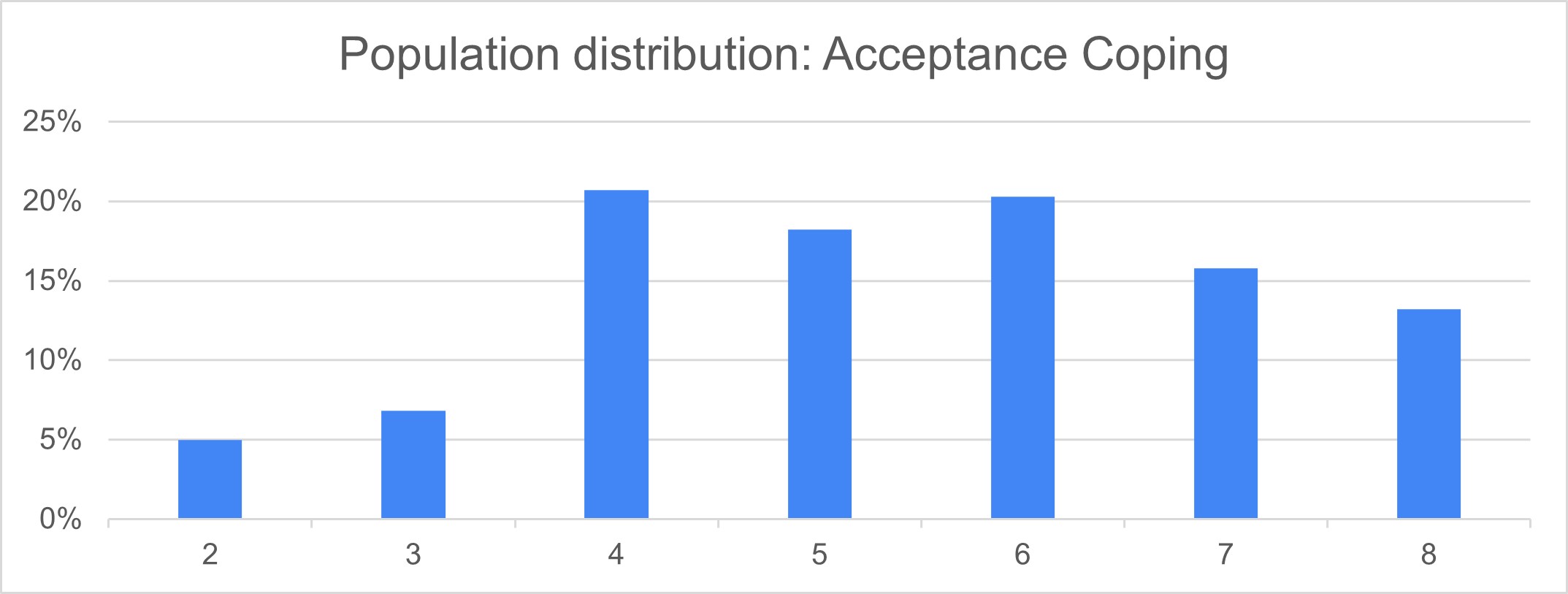

Supplement: Supplementary file 1 [file Data_Sheet_1.zip › Supplementary Figures/Figure 21 (SM). Population distribution of acceptance coping style.jpg]

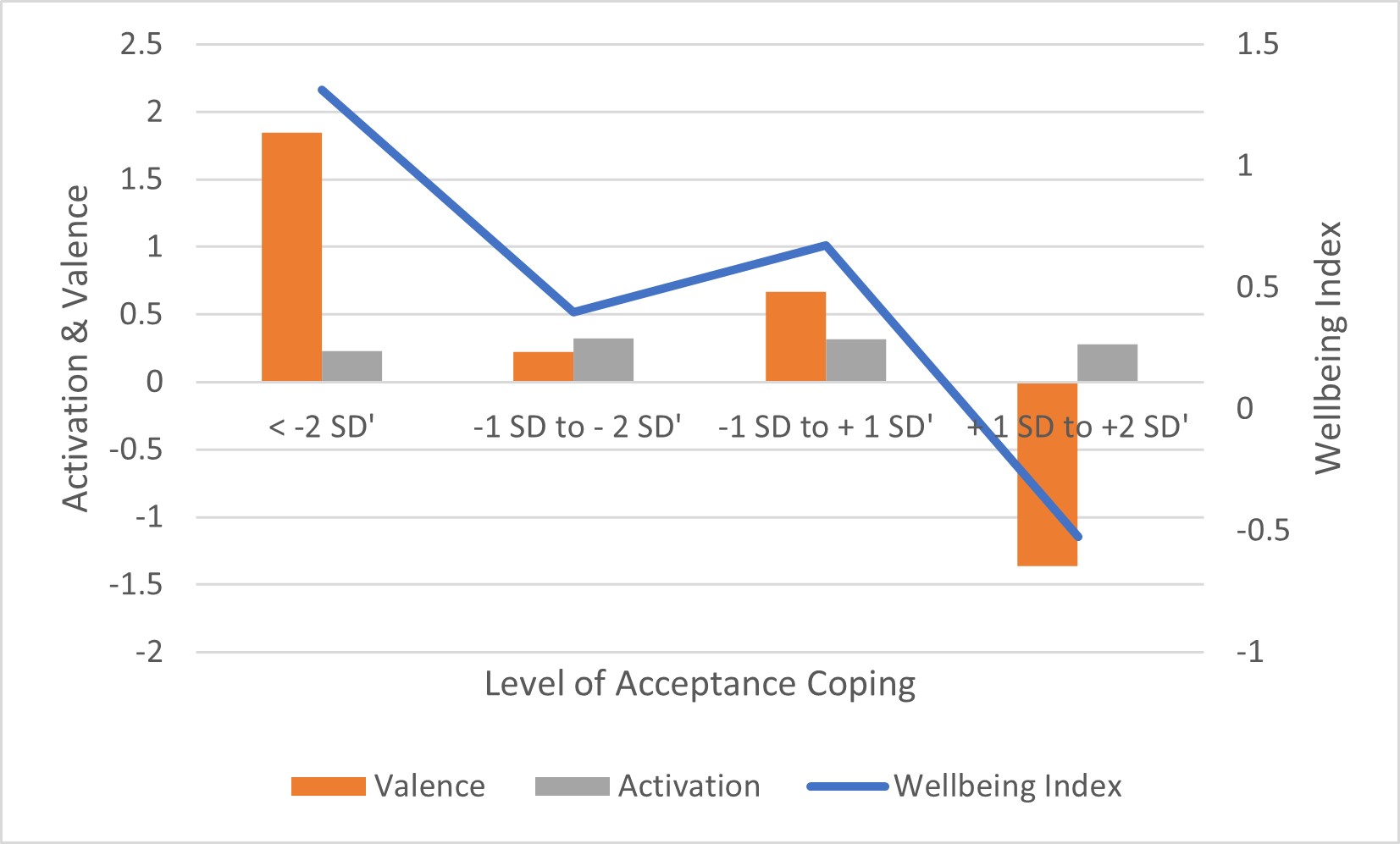

Supplement: Supplementary file 1 [file Data_Sheet_1.zip › Supplementary Figures/Figure 22 (SM). AgileBrain valence, activation, and wellbeing index scores by levels of acceptance coping.jpg]

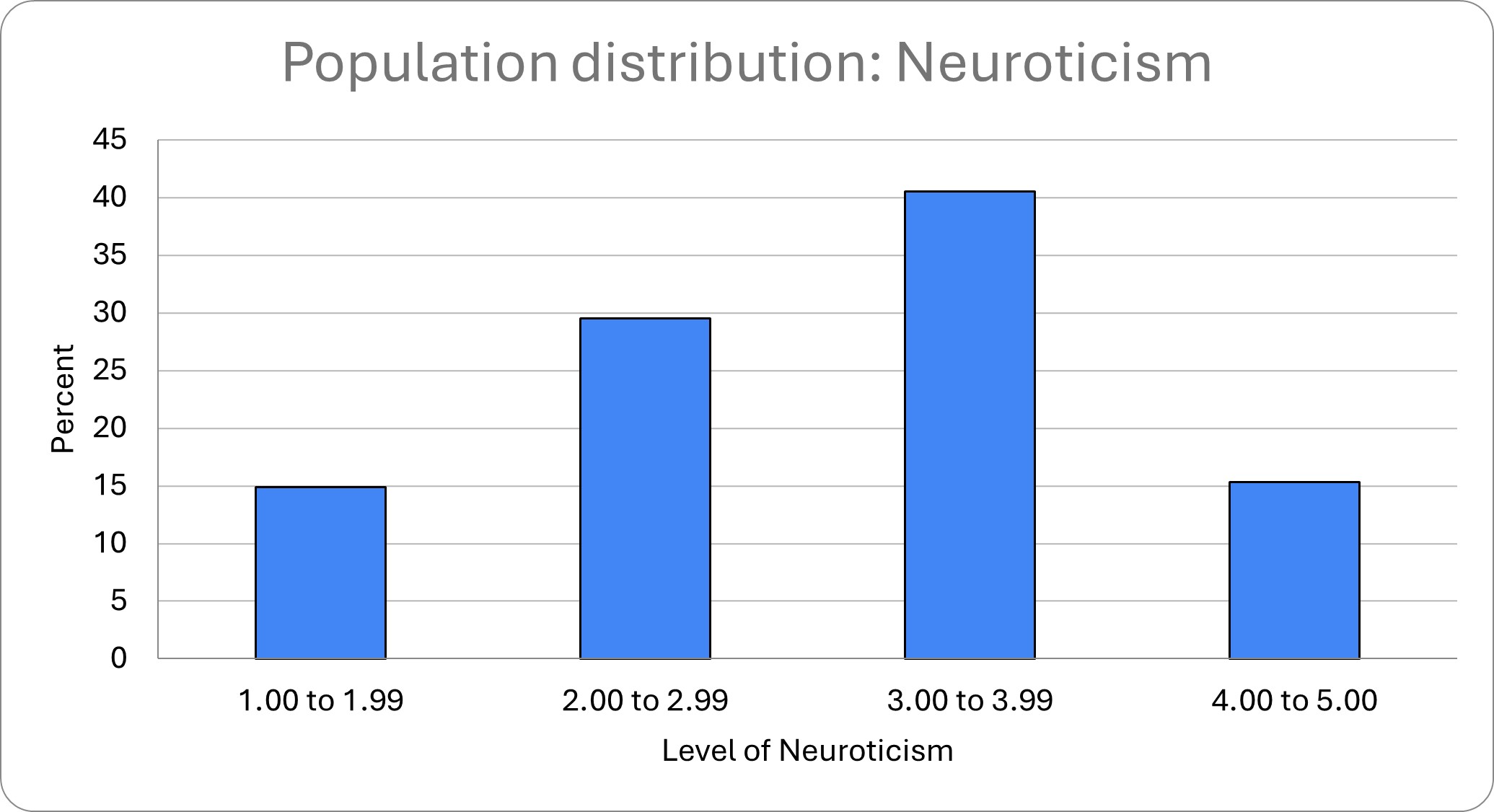

Supplement: Supplementary file 1 [file Data_Sheet_1.zip › Supplementary Figures/Figure 23 (SM). Population distribution of neuroticism .jpg]

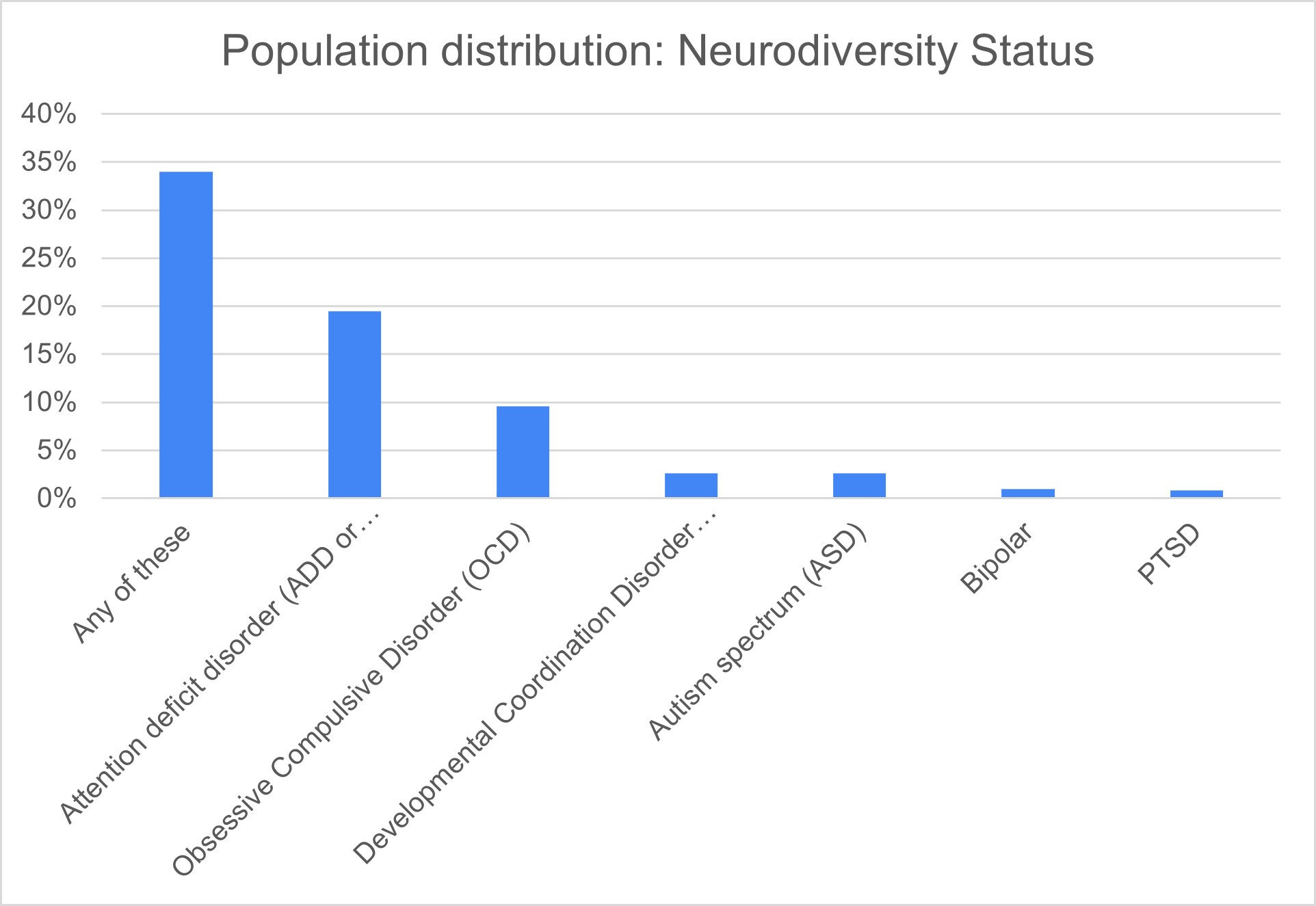

Supplement: Supplementary file 1 [file Data_Sheet_1.zip › Supplementary Figures/Figure 24 (SM). Population distribution of diagnosed neurodiversity .jpg]

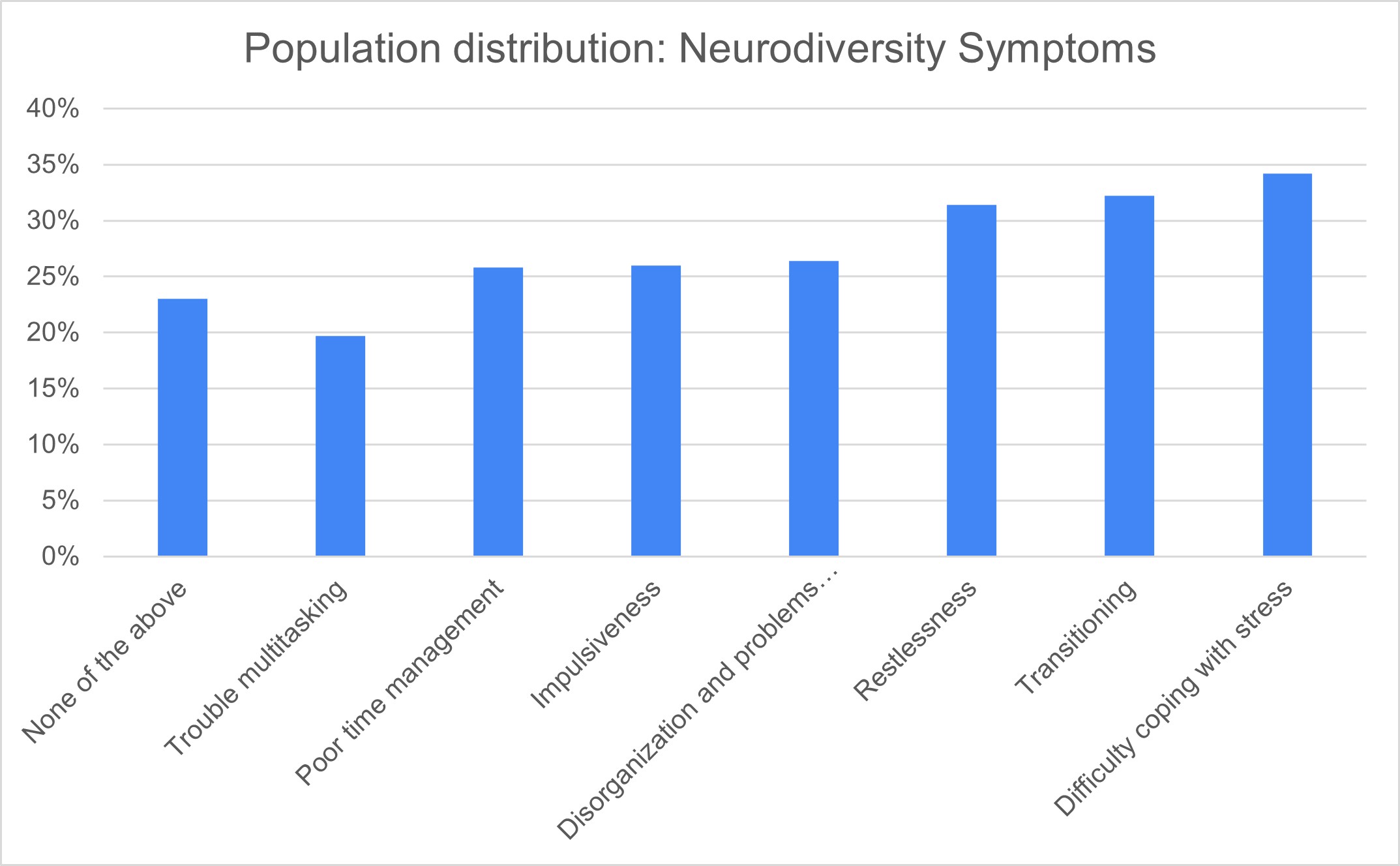

Supplement: Supplementary file 1 [file Data_Sheet_1.zip › Supplementary Figures/Figure 25 (SM). Population distribution of neurodiversity symptoms.jpg]

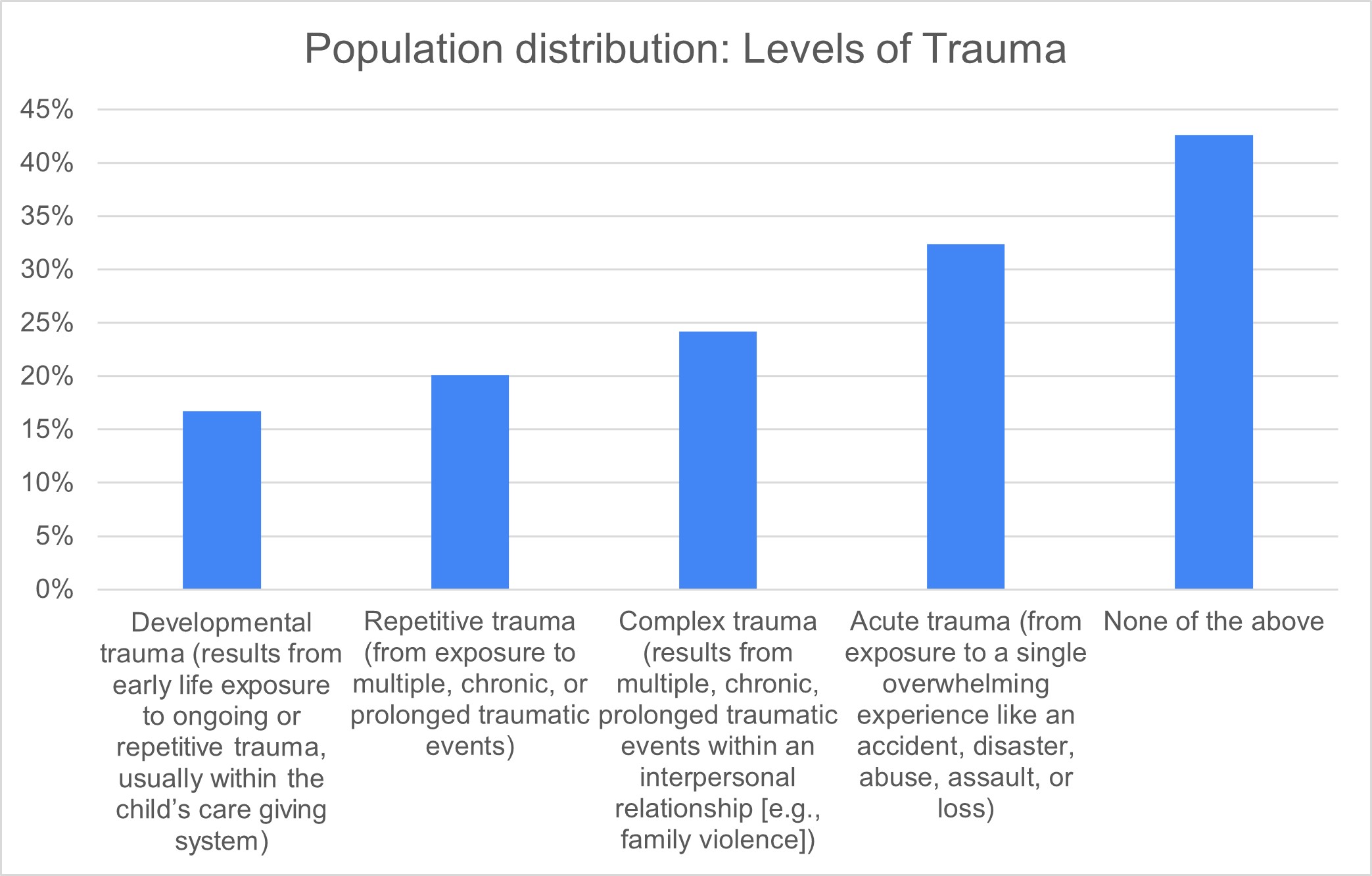

Supplement: Supplementary file 1 [file Data_Sheet_1.zip › Supplementary Figures/Figure 26 (SM). Population distribution of trauma levels .jpg]

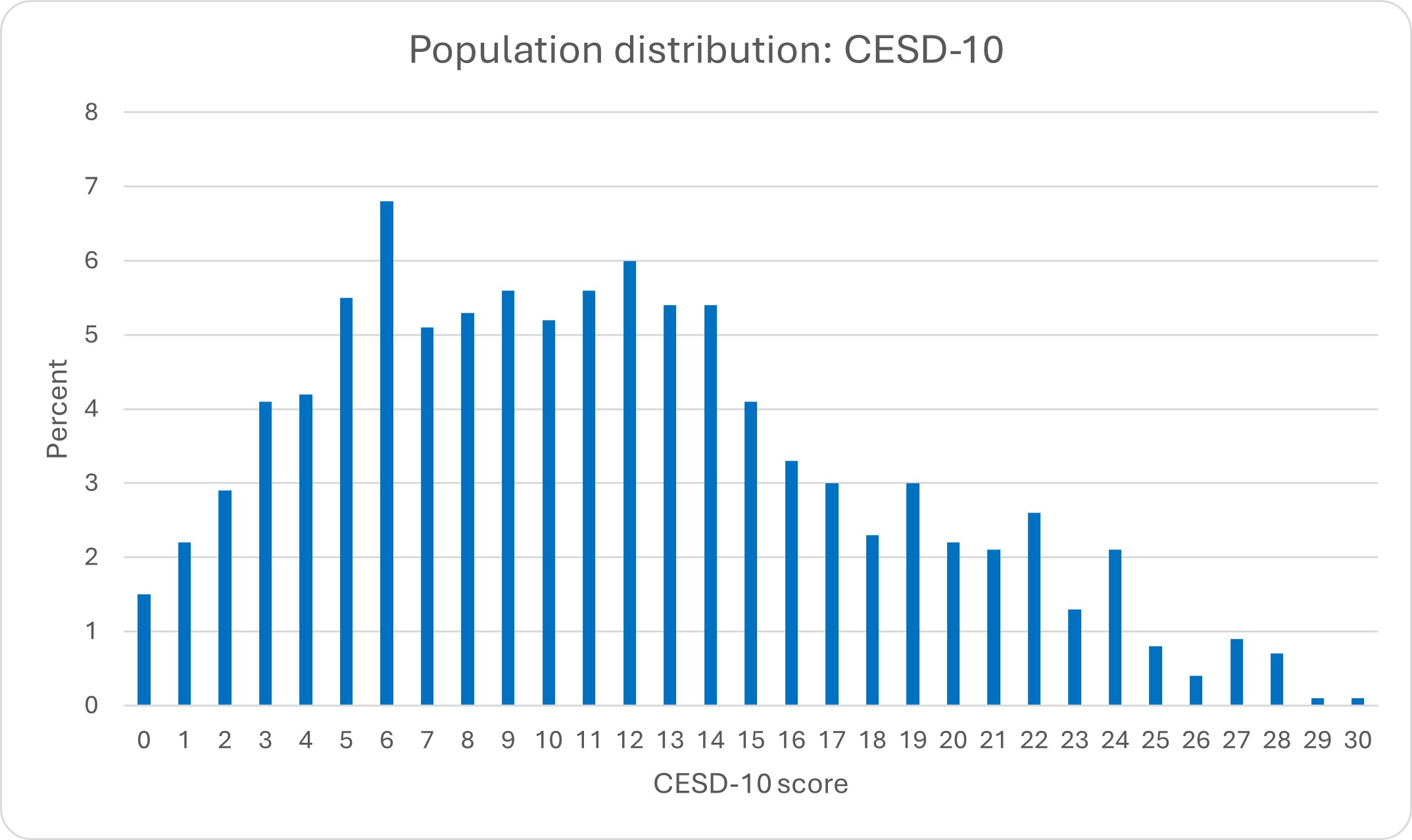

Supplement: Supplementary file 1 [file Data_Sheet_1.zip › Supplementary Figures/Figure 3 (SM). Population distribution of CESD-10 scores.jpg]

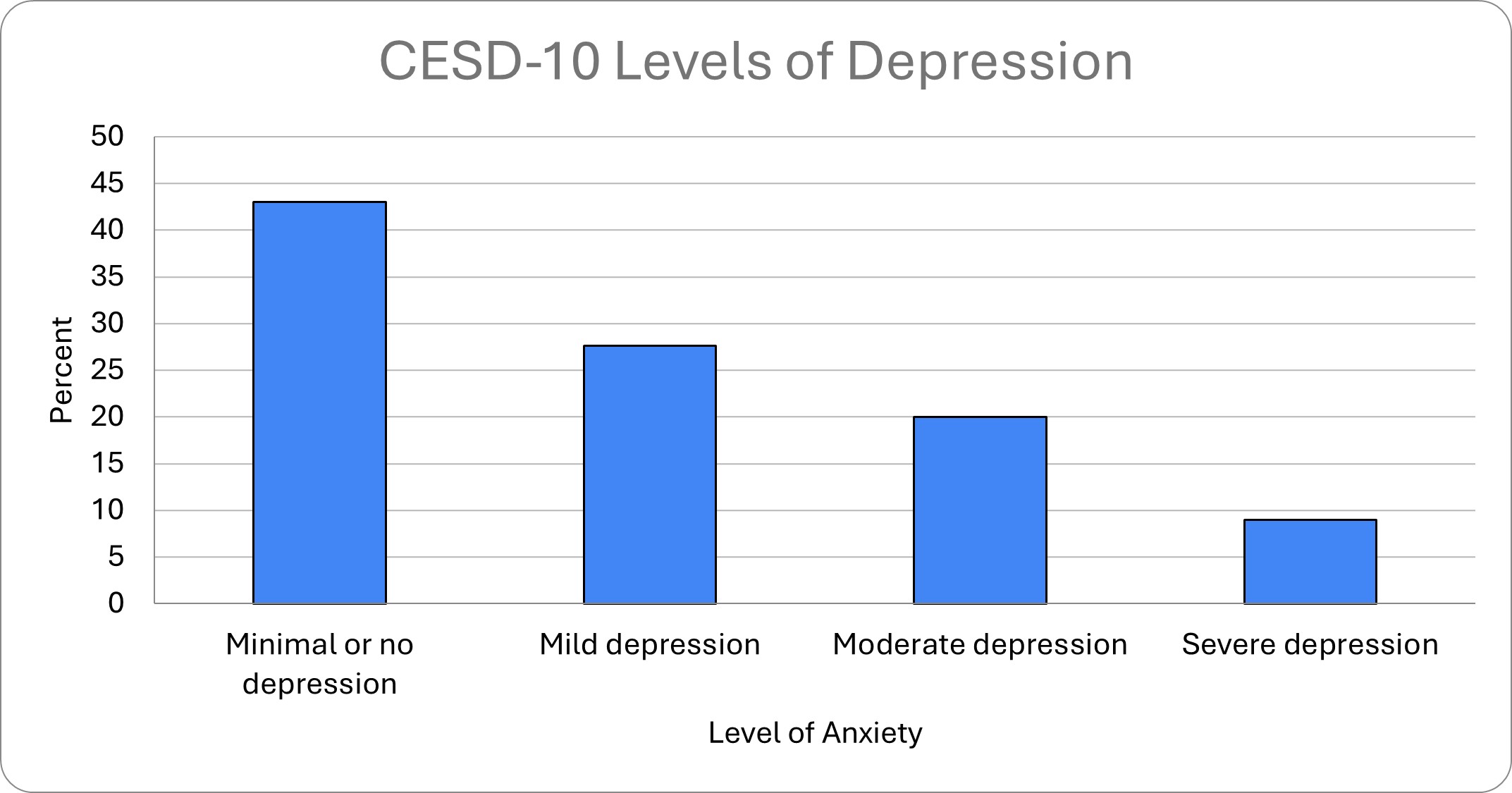

Supplement: Supplementary file 1 [file Data_Sheet_1.zip › Supplementary Figures/Figure 4 (SM). Population distribution of CESD-10 standard levels of depression.jpg]

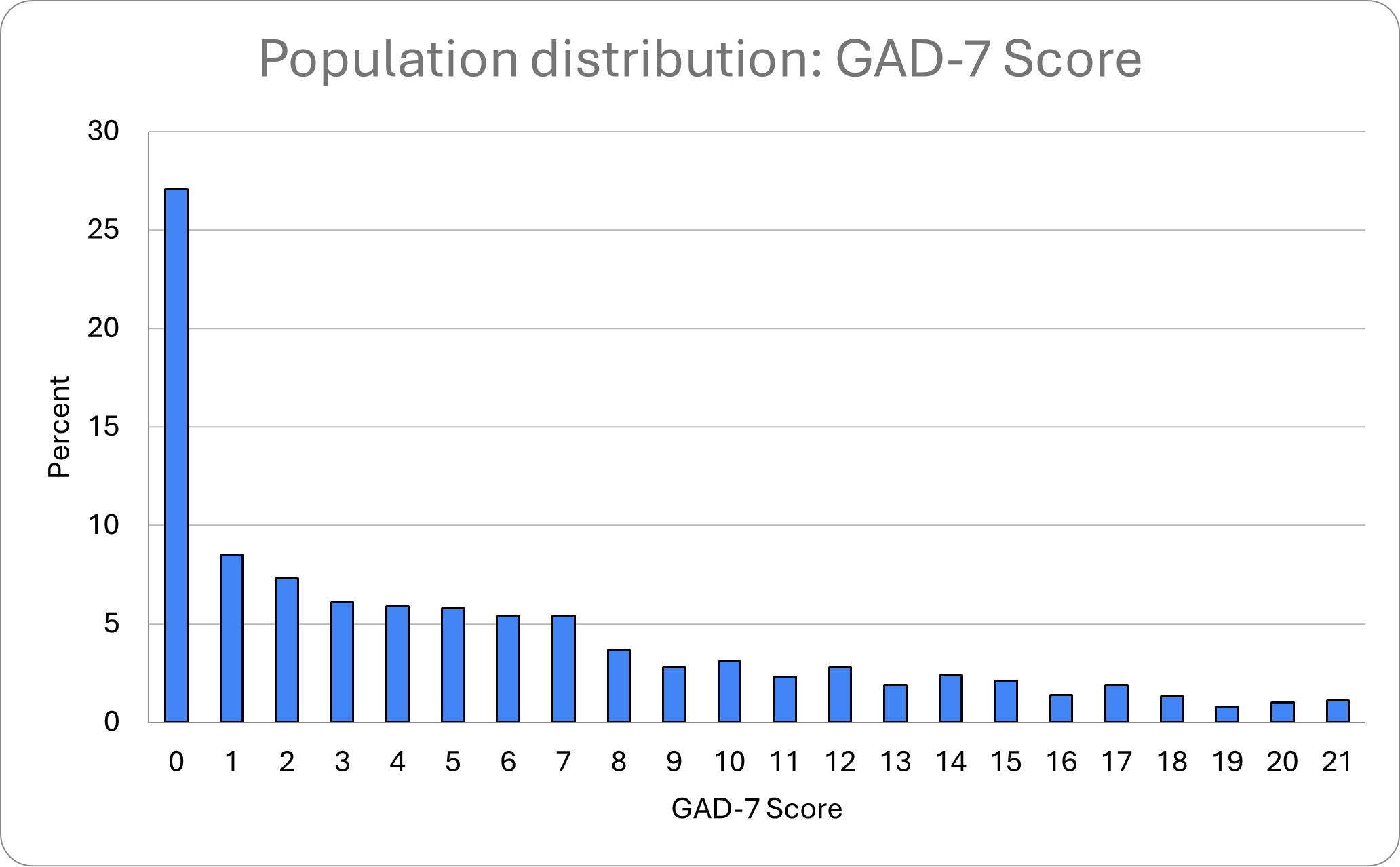

Supplement: Supplementary file 1 [file Data_Sheet_1.zip › Supplementary Figures/Figure 5 (SM). Population distribution of GAD-7 scores.jpg]

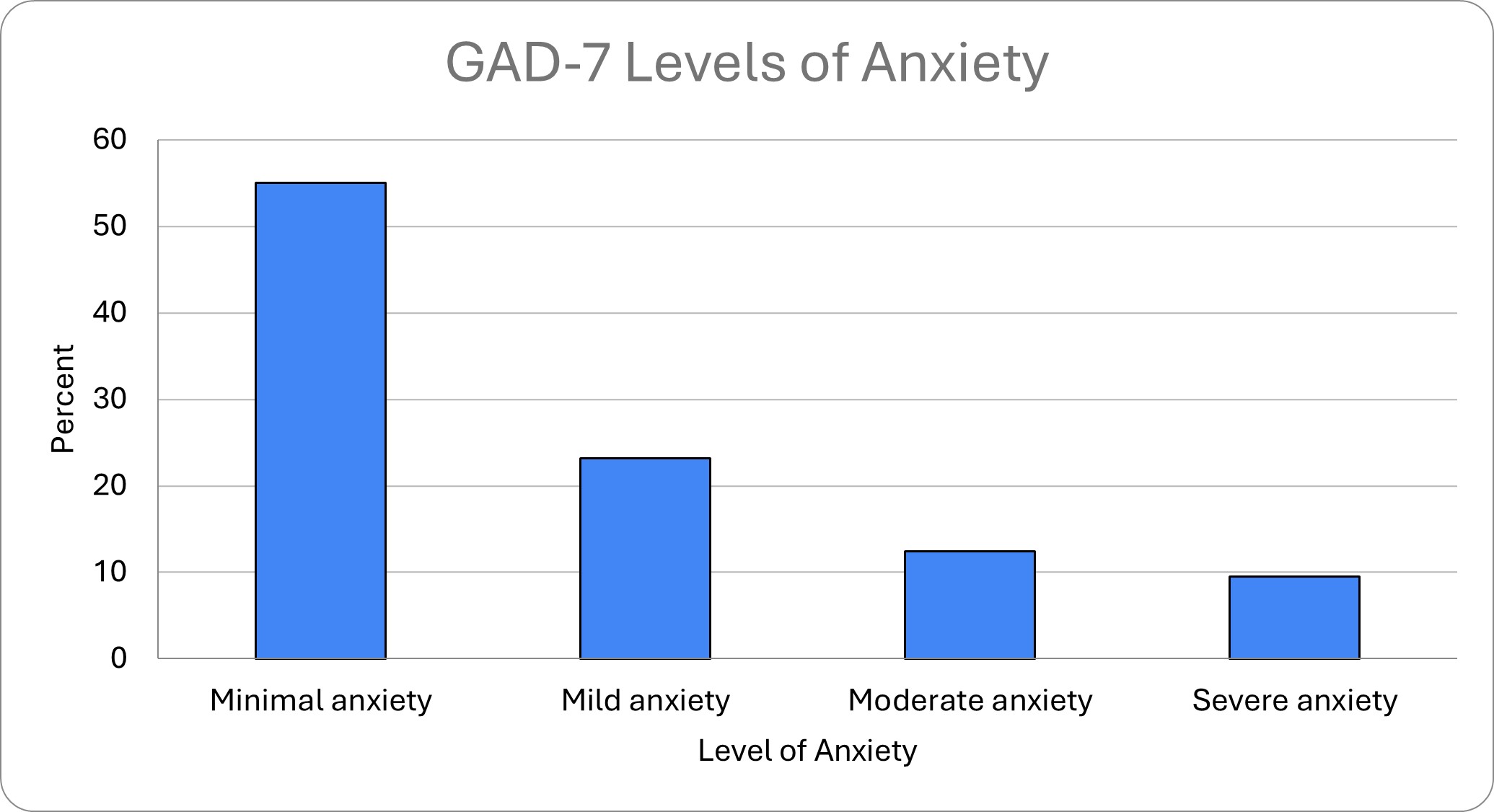

Supplement: Supplementary file 1 [file Data_Sheet_1.zip › Supplementary Figures/Figure 6 (SM). Population distribution of GAD-7 standard levels of anxiety.jpg]

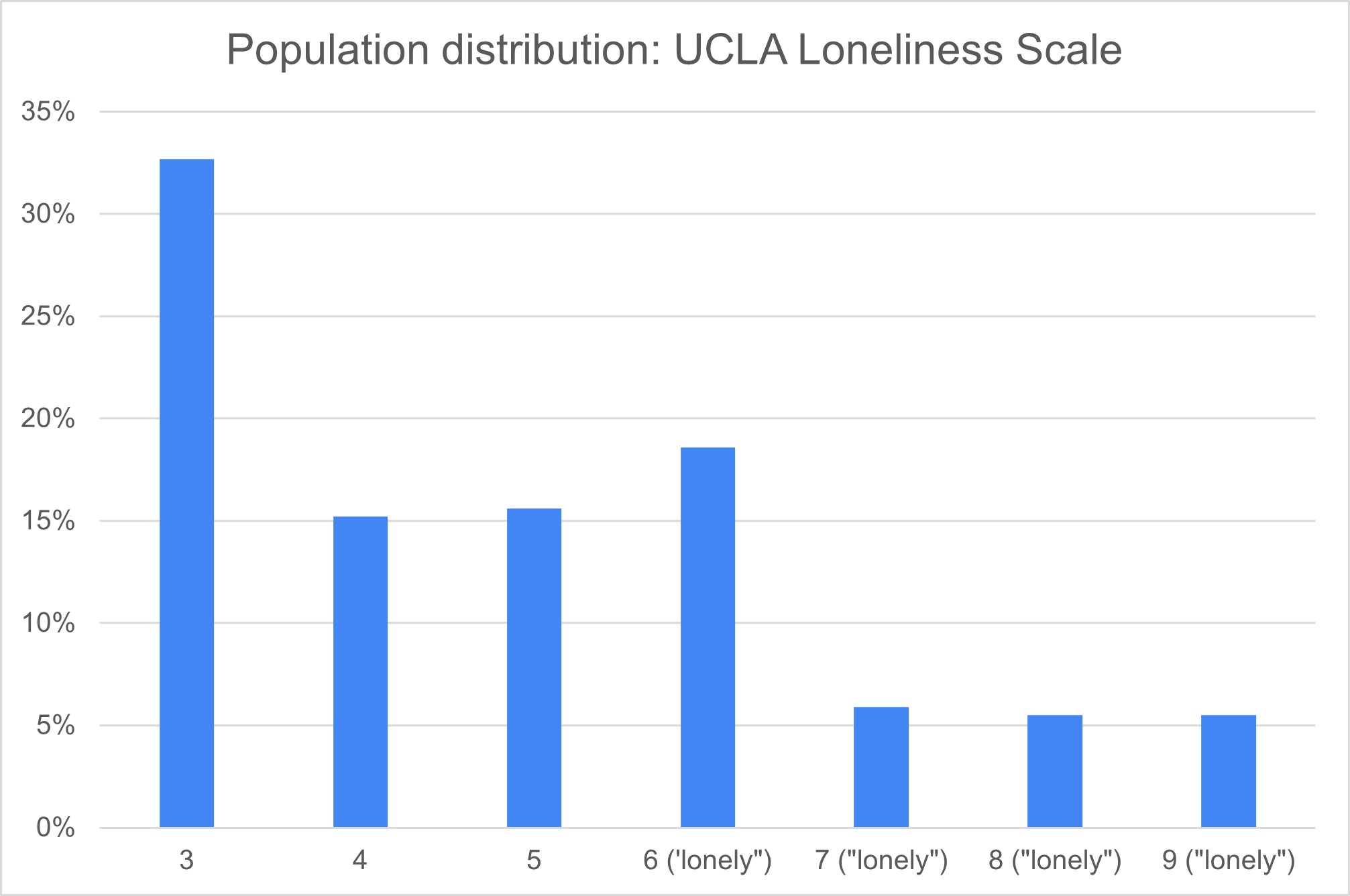

Supplement: Supplementary file 1 [file Data_Sheet_1.zip › Supplementary Figures/Figure 7 (SM). Population distribution of UCLA loneliness scores.jpg]

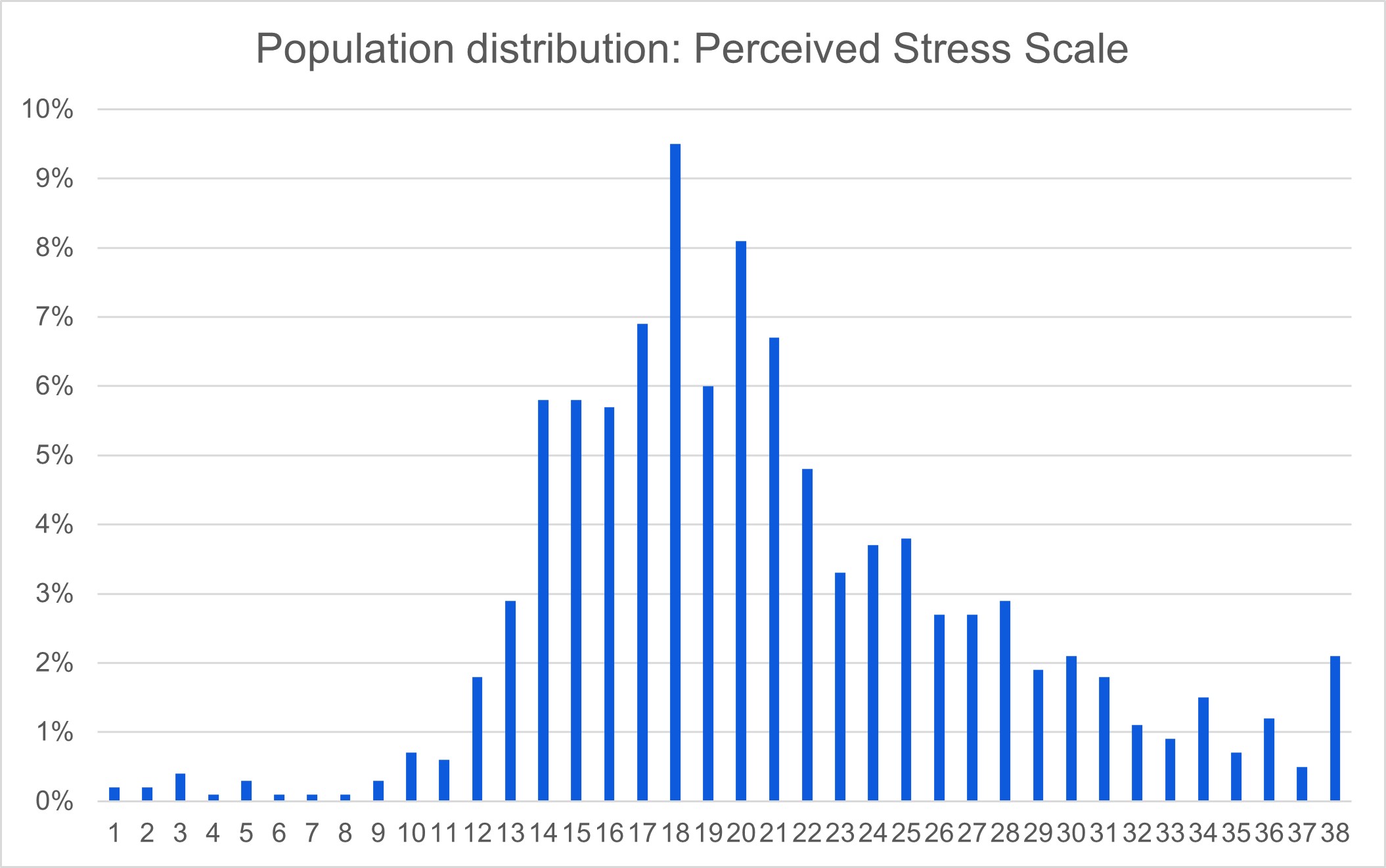

Supplement: Supplementary file 1 [file Data_Sheet_1.zip › Supplementary Figures/Figure 8 (SM). Population distribution of perceived stress.jpg]

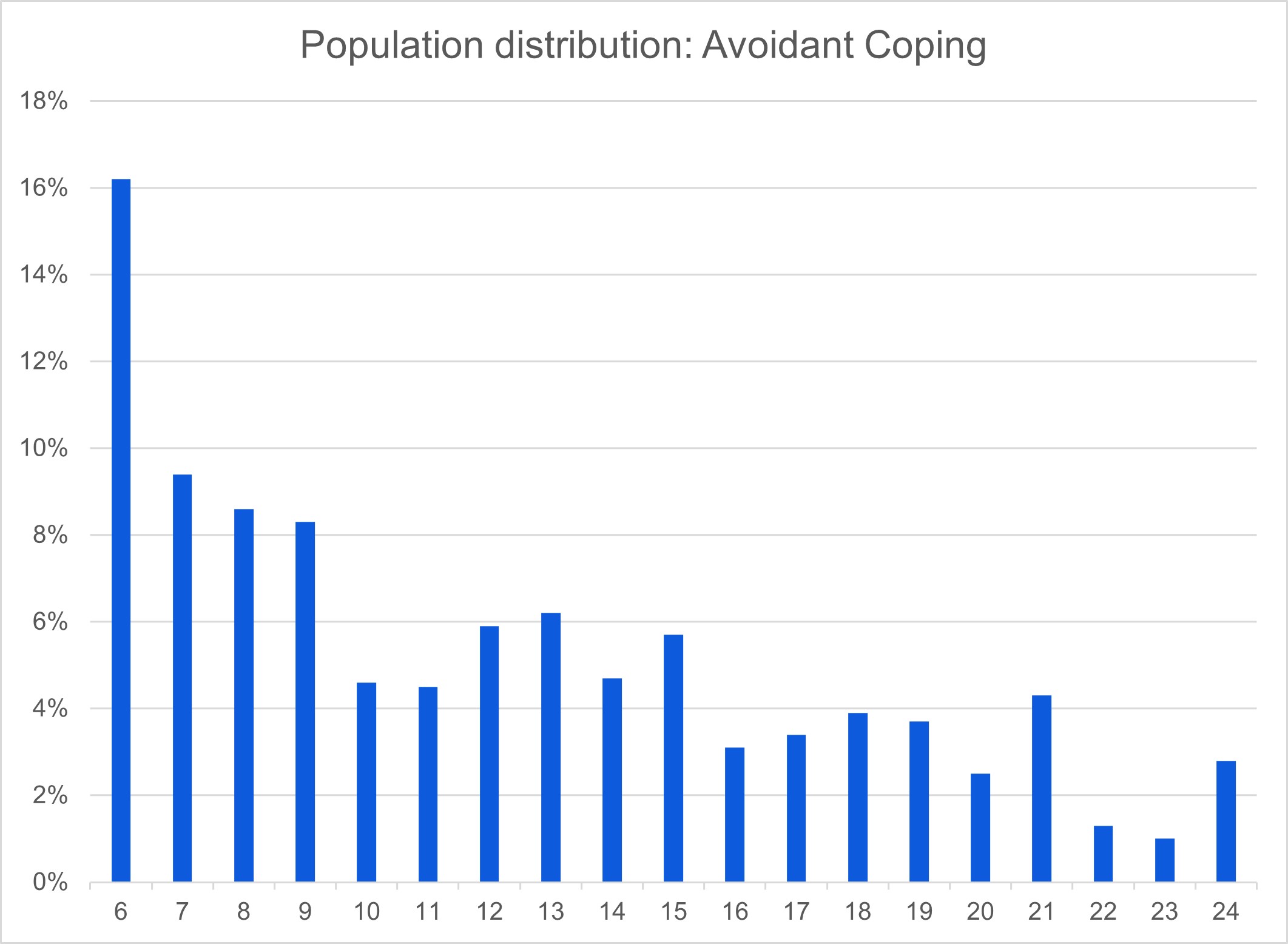

Supplement: Supplementary file 1 [file Data_Sheet_1.zip › Supplementary Figures/Figure 9 (SM). Population distribution of avoidant coping style.jpg]
