# Supplementary material for: Validation of a brief image elicitation task as an indicator of subjective wellbeing in the general population
Source: Front Public Health. 2024 Oct 22;12:1435144. doi: 10.3389/fpubh.2024.1435144 (PMC11534616; doi:10.3389/fpubh.2024.1435144)
Supplement: Supplementary file 2 [file Table_1.docx]

Supplementary tables

Supplementary Table 1. Classification accuracy rates of well-being outcomes using AgileBrain data as predictors using discriminant function analysis

| Classification basis | Method | PHQ-9 (5 levels) | GAD-7 (4 levels) |
| --- | --- | --- | --- |
| Highest vs. lowest level | All cases | 92.2% | 81.2% |
|  | Cross-validated | 75.4% | 76.7% |
| Highest two vs. lowest two levels | All cases | 80.0% | 69.2% |
|  | Cross-validated | 75.1% | 64.0% |
| Upper half vs. lower half | All cases | 70.7% | 69.2% |
|  | Cross-validated | 66.4% | 64.0% |

Supplementary Table 2. Correlation pattern of AgileBrain data with several wellbeing indicators.

|  | AgileBrain summary metrics | | |
| --- | --- | --- | --- |
| Comparison wellbeing indicators | Activation | Valence | Wellbeing index |
| PHQ-9 | + | - | - |
| CESD-10 | + | - | - |
| GAD-7 | + | - | - |
| UCLA-3 | + | - | - |
| PSS-10 | ns | - | - |
| Neuroticism | + | - | - |
| Diagnosed neurodiversity | + | - | - |
| ADHD symptoms | + | - | - |
| Trauma | + | - | - |

Key: + = significant positive correlation; - = significant negative correlation; ns = non-significant

Supplementary Table 3. Theoretical and empirical characteristics of 8 coping styles (Pilch et al., 2021).

| Theoretical adaptiveness | Coping style | Rationale | AgileBrain wellbeing index among users  (+ 1 σ to +2 σ) |
| --- | --- | --- | --- |
| Adaptive | Social support | Seeking help, comfort, and advice is adaptive as it provides emotional relief, practical assistance, and a sense of belonging | +0.29 |
|  | Spirituality | Can provide meaning, comfort, and a sense of community, which can enhance resilience and emotional well-being | +0.13 |
|  | Problem-focused | Involves directly addressing and managing the stressor, aiming to resolve root causes​ | -0.22 |
|  | Acceptance | Acknowledging the reality of a situation and coming to terms with it can reduce emotional distress and help focus on controllable aspects | -0.84 |
| Mixed | Distraction | Distraction can be adaptive in the short term to prevent overwhelm but becomes maladaptive if it prevents addressing the root causes of stress | +0.11 |
| Maladaptive | Externalizing | Blaming others or external factors for one's problems prevents personal growth and can lead to conflicts and unresolved issues | -0.59 |
|  | Avoidant | Can lead to unresolved issues and increased stress over time | -0.60 |
|  | Self-blame | Promotes guilt, shame, and lowered self-esteem, which can exacerbate stress and hinder effective problem-solving​ | -1.58 |
